# IL-24 Inhibits Lung Cancer Growth by Suppressing GLI1 and Inducing DNA Damage

**DOI:** 10.3390/cancers11121879

**Published:** 2019-11-27

**Authors:** Janani Panneerselvam, Akhil Srivastava, Meghna Mehta, Allshine Chen, Yan D. Zhao, Anupama Munshi, Rajagopal Ramesh

**Affiliations:** 1 Department of Pathology, The University of Oklahoma Health Sciences Center, Oklahoma City, OK 73104, USA; janani-panneerselvam@ouhsc.edu (J.P.); akhil-srivastava@ouhsc.edu (A.S.); 2 Stephenson Cancer Center, The University of Oklahoma Health Sciences Center, Oklahoma City, OK 73104, USA; meghna-mehta@ouhsc.edu (M.M.); allshine-chen@ouhsc.edu (A.C.); daniel-zhao@ouhsc.edu (Y.D.Z.); anupama-munshi@ouhsc.edu (A.M.); 3 Department of Radiation Oncology, The University of Oklahoma Health Sciences Center, Oklahoma City, OK 73104, USA; 4 Department of Biostatistics and Epidemiology, The University of Oklahoma Health Sciences Center, Oklahoma City, OK 73104, USA; 5 Graduate Program in Biomedical Sciences, The University of Oklahoma Health Sciences Center, Oklahoma City, OK 73104, USA

**Keywords:** lung cancer, IL-24, GLI1, DNA damage, apoptosis, hedgehog signaling

## Abstract

Aberrant expression of GLI1 is responsible for aggressive tumor behavior and survival due to its effects on the DNA damage response (DDR). We investigated whether interleukin (IL)-24, a tumor suppressor, inhibits GLI1 and the associated DDR pathway in human NSCLCs. IL-24 treatment reduces mRNA and protein expression of GLI1 in lung tumor cells, but not in normal cells. GLI1 reporter assay and mRNA studies demonstrated that IL-24 regulates GLI1 at the post-transcriptional level by favoring mRNA degradation. Associated with GLI1 inhibition was marked suppression of the ATM-mediated DDR pathway resulting in increased DNA damage, as evidenced by γ-H2AX foci and Comet assay. Furthermore, attenuation of GLI1-associated DDR by IL-24 increased caspase-3 and PARP activity, resulting in cancer cell apoptosis. GLI1 inhibition and overexpression confirmed that IL-24-mediated anti-tumor effects involved the GLI-dependent pathway. Finally, we observed that IL-24-mediated alteration in GLI1 is independent of the canonical hedgehog-signaling pathway. Our study provides evidence that IL-24 treatment induces DNA damage, and reduces GLI1 expression and offers an opportunity for testing IL-24-based therapy for inhibiting GLI1 in lung cancer.

## 1. Introduction

Lung cancer remains the leading cause of cancer-related death worldwide, despite extensive advances made in treatment approaches [1,2,3,4,5]. Drug resistance and metastasis make effective treatment strategies difficult [6]. Several ongoing studies into the molecular basis of lung cancer have shown that various signaling pathways are deregulated during the tumorigenic process and are involved in the resistance of cancer cells to existing therapies [7]. Therefore, novel biological therapies targeting oncogenic signaling pathways that initiate andeffectively kill tumor cells will improve the overall survival of patients with lung cancer.

Sonic hedgehog (SHH) is one of the major developmental pathways and regulates embryonic development, tissue homeostasis, and cell fate in vertebrate and invertebrate organisms [8]. However, aberrant activation and deregulation of SHH signaling lead to tumor initiation and progression, and promote cancer stemness, epithelial-mesenchymal transitions (EMT), drug resistance, invasion of surrounding tissues, and metastasis to distant organs for secondary tumor formation in multiple tumor types [9,10]. Glioma-associated oncogene homolog 1 (GLI1) is a transcription factor and is the main downstream target of SHH signaling [11]. Overexpression of GLI1 has been reported in multiple solid tumor types, including medulloblastoma [12], rhabdomyosarcoma [13,14], biliary [15], breast cancer [16,17,18], prostate cancer [19,20] colon cancer [21], bladder cancer [22], and lung cancer [23], and is also associated with metastatic tumors [15,18,19]. SHH signaling activates GLI1 by initiating the binding of SHH ligand to a 12-transmembrane receptor PATCHED (PTCH), resulting in the activation of the smoothened (SMO) seven trans-membrane domain protein. Activated SMO induces a series of intracellular events, which in turn activate downstream target genes through the zinc-finger transcription factors GLI1, GLI2, and GLI3. Recent studies demonstrated that GLI1 can also be activated by non-canonical pathways—such as PI3K/AKT, MAPK, WNT, NF-κB, K-RAS, and TGFβ—and appears to be an integrative platform of various signaling inputs [11,24].

Studies have shown that among the three human GLI factors, GLI1 and GLI2 are crucial for the development and progression of many human cancers, including lung cancer [25]. Overexpression of GLI1 plays a crucial role in the ATM-mediated DNA damage response (DDR) signaling, anti-apoptosis, drug resistance, EMT, and cancer stemness, resulting in cancer cell survival and progression [26]. Preclinical studies demonstrated that pharmacological and genetic inhibition of HH/GLI1 signaling resulted in anticancer activity [25]. However, results from clinical trials are not encouraging, due to resistance and non-specific toxicity. These inhibitors are limited to only certain tumors and are largely ineffective in others [11,27]. Thus, new and improved therapeutics targeting GLI1 are warranted.

IL-24 previously referred to as human melanoma differentiation-associated gene (mda)-7 is a member of the IL-10 cytokine family [28]. Prior studies have reported IL-24 protein expression is lost in a broad-spectrum of human cancer cell lines and its expression correlated with disease prognosis in melanoma and lung cancer [28,29,30,31,32]. Furthermore, restoring IL-24 protein expression using viral and non-viral-based gene IL-24 delivery resulted in suppression of tumor growth, angiogenesis, and metastasis both in vitro and in vivo [33,34,35]. The results from all of these studies supported the tumor suppressor properties of IL-24 and resulted in the testing of adenovirus-mda-7 (INGN-241)-based cancer gene therapy in a phase I clinical trial for solid tumors [36]. Whilst IL-24 as a cancer therapeutic is accepted, its role on HH signaling and/or its downstream target GLI1 remains elusive.

In the present study, we investigated the ability of IL-24 to inhibit GLI1 expression and its associated DDR pathway. The rationale for the present investigation is that prior studies from our laboratory showed that IL-24 inhibits the AKT-mTOR and SDF-1/CXCR4 signaling axis [33,35], which are major pathways regulating GLI1 activation [13,26]. Hence, we hypothesized that IL-24 suppresses GLI1 and induces DNA damage in lung tumor cells leading to cell death.

## 2. Results

### 2.1. GLI1 Expression in Lung Adenocarcinoma 

**Figure 1:**
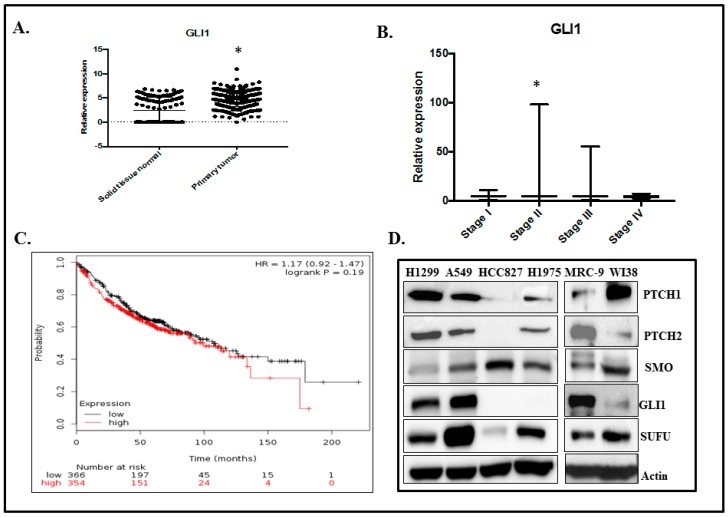
GLI1 expression in human lung adenocarcinoma. (**A**) The TCGA LUAD database of 577 patients showed that GLI1 mRNA expression is higher (* Represents *p* < 0.05) in the primary tumor samples than in normal solid tissues. (**B**) The pathological stage of the LUAD dataset demonstrated that GLI1 mRNA expression is highly elevated in Stage II and III lung adenocarcinoma, compared with Stage I and IV lung adenocarcinoma. However, a significant increase in GLI1 was observed in Stage II but not in Stage III when compared to Stage I (*p* < 0.013). No significant difference in GLI1 mRNA was observed between Stage I and IV. (**C**) Kaplan–Meier plot of 720 lung cancer patients analyzed from GEO, EGA, and TCGA data bases showed that patients with high GLI1 gene expression had low overall survival compared with patients with low GLI1 expression (*p* = 0.1932). (**D**) Hedgehog signaling proteins expression in human lung cancer (H1299, A549, HCC827, H1975) and normal lung (MRC-9, WI38) cell lines.

To assess whether GLI1 plays a role in lung adenocarcinoma, we generated a GLI1 gene expression dataset from the TCGA LUAD database of 577 patients. We observed that GLI1 mRNA expression is higher in the primary tumor samples than in normal solid tissues (Figure 1A; *p* < 0.05). GLI1 expression in the pathological stage of the LUAD dataset demonstrated that GLI1 is elevated in stage II and III lung adenocarcinoma compared with stage I and IV lung adenocarcinoma. However, a significant increase in GLI1 mRNA expression was observed in stage II but not in stage III when compared to stage I disease (*p* < 0.013). There was no significant difference in GLI1 between stage I and IV. This data suggests that GLI1 may be a requirement in the early stages for driving lung cancer progression (Stage II and III) and not a requirement at late stage (IV) (Figure 1B). Kaplan–Meier survival curve analysis of 720 lung cancer patients showed that patients with high GLI1 gene expression had a trend towards having low overall survival compared with patients with low GLI1 expression (Figure 1C; *p* = 0.1932). However, there was no statistical significance between the two groups analyzed.

We next analyzed the expression levels of canonical SHH signaling components—such as PTCH1, PTCH2, SMO, SUFU, and GLI1 proteins—in cultured human lung cancer cells (H1299, A549, HCC827, and H1975) and normal human lung fibroblasts (MRC-9 and WI38) by western blot analysis. The expression levels of SHH signaling proteins varied among the cancer cell lines and normal cell lines (Figure 1D). Based on the GLI1 expression data, H1299 and A549 cancer cell lines and MRC-9 normal cells were chosen for the present study.

### 2.2. GLI1 Expression Is Reduced in H1299-IL24 Cells

**Figure 2:**
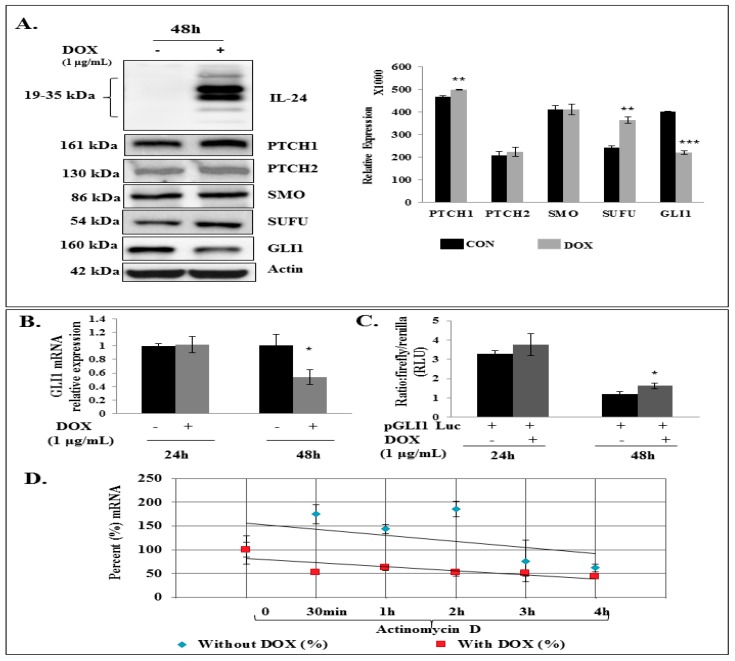
IL-24 reduced GLI1 expression in H1299-IL24 lung cancer cells. (**A**) IL-24 reduced GLI1 expression, with increases in PTCH1 and SUFU, at 48 h in H1299-IL24 cells compared with control cells. (**B**) RT-PCR analysis showed that IL-24 reduced GLI1 mRNA levels at 48 h. (**C**) GLI promoter activity was determined using a luciferase reporter vector. Induction of IL-24 showed no significant change in luciferase activity, indicating that IL-24 did not affect GLI at the promoter level. (**D**) mRNA stability studies showed that IL-24 reduced the half-life of GLI1 mRNA approximately at 30 min. The gene expression was standardized using 18S as a reference gene. Differences in the expression of the proteins were determined by semi-quantitative analysis and represented in graphical format. Each experiment was performed at least two times. * Represents *p* < 0.05, ** represents *p* < 0.001, *** represents *p* < 0.0001, ns = not significant. Bars denote standard deviation (SD).

To determine the effect of the tumor suppressor IL-24 on the expression of SHH signaling components, we used the H1299 cell line (labeled ‘H1299-IL24’), which was stably transfected with doxycycline-inducible plasmid vector (pTET-IL-24), as described previously [37]. H1299-IL24 cells were treated with 1µg/mL doxycycline to express IL-24. Induction of IL-24 expression produced no marked change in the levels of PTCH2 and SMO (Figure 2A). However, we observed an increase in the expression of PTCH1 and SUFU (Figure 2A; *p* < 0.001) compared with control cells that were not induced to express IL-24. Notably, induced expression of IL-24 showed a significant reduction in GLI1 protein expression (Figure 2A; *p* < 0.0001) at 48 h. We observed a similar trend in reduced SUFU and GLI1 protein expression at 72 h (Supplementary Figure S1).

Next, to ascertain whether IL-24 inhibits GLI1 at the mRNA level, H1299-IL24 cells were induced to express IL-24. Expression of IL-24 significantly reduced GLI1 mRNA expression at 48 h compared with control (Figure 2B; *p* < 0.05) suggesting that IL-24 regulates GLI1 protein expression by reducing it at the mRNA level. In order to demonstrate the mechanism by which IL-24 regulates GLI1 mRNA expression, we analyzed the promoter activity in H1299-IL24 cells that were transiently transfected with a luciferase reporter vector driven by the human GLI promoter region. Our results show that induction of IL-24 failed to inhibit GLI promoter activity, as shown by the lack of reduction in luciferase reporter activity (Figure 2C). This reveals that IL-24 did not regulate GLI mRNA expression at the promoter level. Next, we pretreated the cells with doxycycline for 48 h and subsequently treated them with or without (control) the transcription inhibitor actinomycin D. Cells were collected after actinomycin D treatment at different time points to analyze the expression levels of GLI1 mRNA. We observed that the GLI1 mRNA level was significantly reduced (>3 fold reduction over control; *p* < 0.001) by IL-24 expression starting from 30 min to 2 h of actinomycin D treatment when compared with control cells (Figure 2D). This finding clearly indicates that IL-24 decreases GLI1 mRNA post-transcriptionally by affecting its stability.

It has been reported that GLI1 and GLI2 play important roles in tumor development and progression [25]. Hence, we also analyzed the inhibitory effect of IL-24 on GLI2 expression. We observed that expression of IL-24 did not affect GLI2 at the mRNA level. However, a marginal decrease in GLI2 protein expression was observed at 48 h (*p* < 0.01), but not at 72 h (Supplementary Figure S2).

To determine whether the observed IL-24-mediated downregulation of GLI1 was restricted to one cell line with a stable transfection system, we performed experiments in an additional lung cancer cell line, A549. A549 and H1299 cells were transiently transfected with an IL-24-expressing plasmid DNA vector [35]. The cells were harvested and cell lysates were analyzed by RT-PCR and western blotting (Supplementary Figure S3). Expression of IL-24 in H1299 cells significantly reduced GLI1 mRNA (*p* < 0.05) and protein expression at 48 h (*p* < 0.05), compared with control cells that did not express IL-24. In addition, an increase in the expression of SUFU and gamma-H2AX was observed (*p* < 0.05). Similarly, transfection of IL-24 in A549 cells showed reduction in GLI1 mRNA and protein expression (*p* < 0.05), and was associated with an increase in the expression of SUFU and gamma-H2AX (*p* < 0.05) (Supplementary Figure S3). This finding shows that suppression of GLI1 by IL-24 is not exclusive to one cell line, and that IL-24-mediated inhibitory activity on GLI1 is observed using both, inducible and transient expression of IL-24.

We also tested the impact of exogenous expression of IL-24 on SHH signaling proteins in normal human lung fibroblasts (MRC-9). We observed an increase in PTCH2 and GLI1 protein expression but not in PTCH1 expression (Supplementary Figure S4; *p* < 0.05) in IL-24-expressing cells compared with controls. A slight reduction in SMO was observed upon IL-24 expression. These results demonstrated that IL-24 selectively inhibits GLI1 in tumor cells, but not in normal cells.

### 2.3. IL-24 Regulates ATM-DDR Pathway in Lung Cancer Cells

**Figure 3:**
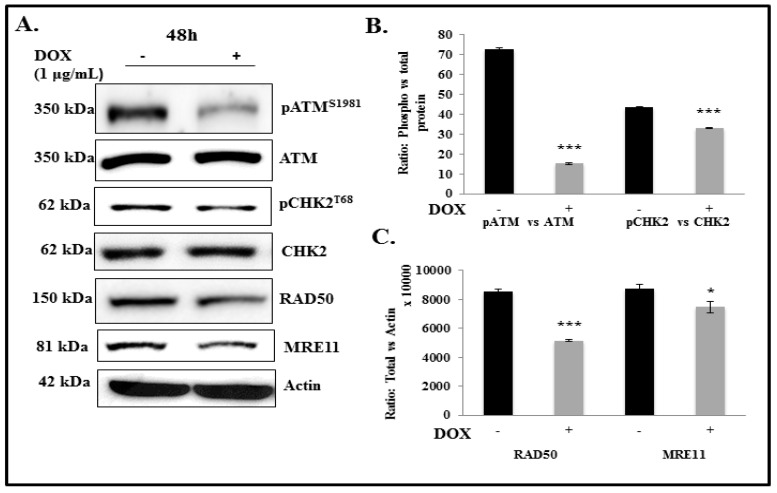
Effect of IL-24 on DDR proteins in H1299-IL24 lung cancer cells. (**A**) Western blotting analysis showed that induction of IL-24 protein in H1299-IL24 cells reduced the expression of phosphorylated (p) ATM^S1981^ and pChk2^T68^, RAD50, and MRE11 at 48 h after doxycycline treatment. Beta-actin was used as a protein loading control. (**B**,**C**) Differences in the expression of the proteins were determined by semi-quantitative analysis and represented in graphical format. The experiment was performed at least two times. * Represents *p* < 0.05, *** represents *p* < 0.0001, ns = not significant. Bars denote standard deviation (SD).

The ATM-DDR pathway is a highly integrated and interconnected pathway that can trigger a variety of cellular responses, including DNA repair, cell cycle arrest, and apoptosis. It is an essential physiological mechanism that maintains genomic integrity in nucleated cells [38]. Studies have demonstrated the important role of GLI1 overexpression in the deregulation of the DDR and repair signaling in cancer cells for their survival during oncogenic stress and to develop chemoresistance [26]. As a consequence of GLI1 inhibition by IL-24, we observed a marked reduction in pATM (*p* < 0.0001) at 48 h compared with controls (Figure 3). Reduction in pCHK2 (*p* < 0.0001), RAD50 (*p* < 0.0001), and MRE11 (*p* < 0.05) expression was also observed albeit less than that observed for pATM (Figure 3). We observed a similar trend of results even at 72 h after IL-24 induction in the H1299-IL-24 cell line (Supplementary Figure S5). This is a clear indication that IL-24 modulates the ATM-mediated DDR pathway in lung cancer cells.

### 2.4. IL-24 Induces DNA Damage in H1299 Lung Cancer Cells

**Figure 4:**
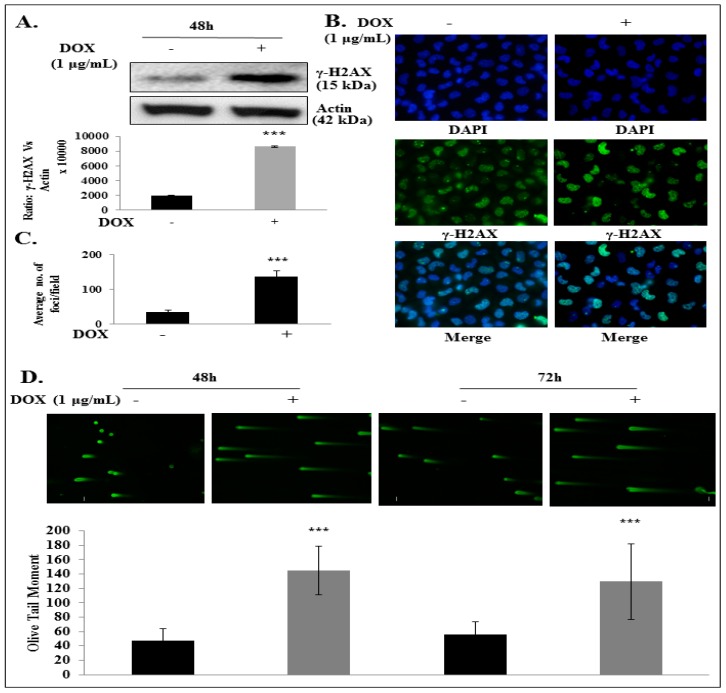
IL-24 induces DNA damage in H1299-IL24 lung cancer cells. (**A**) Western blotting showed that IL-24 expression produced a greater increase in γ-H2AX expression than did control. (**B**) IL-24 increased the number of γ-H2AX foci in DOX-treated H1299-IL24 cells compared with untreated H1299-IL24 cells. (**C**) Quantification of γ-H2AX foci in control and IL-24-induced cells. (**D**) COMET assay showed that DOX-induced IL-24 expression in H1299-IL24 cells increased the Olive tail moment when compared with control. Beta-actin was used as a protein loading control. Differences in the expression of the proteins were determined by semi-quantitative analysis and represented in graphical format. The experiment was performed at least two times. *** represents *p* < 0.0001, ns = not significant. Bars denote standarddeviation (SD).

Studies have shown that pharmacological and genetic inhibition of GLI1 expression in cancer cells induced increased DNA damage and cell death [39,40,41,42]. In the present study, we observed a significant increase in the expression of γ-H2AX, a sensitive marker of double-strand breaks [21], at 48 h and 72 h (*p* < 0.001) in IL-24-expressing H1299 cells compared with controls (Figure 4A; Supplementary Figure S6). We performed qualitative and quantitative evaluation of the DNA damage by immunofluorescence staining of the γ-H2AX foci (Figure 4B,C). It is known that the number of γ-H2AX foci is proportional to the amount of DNA strand breaks. After induction of IL-24 in H1299-IL24 cells, we observed significant changes in cellular morphology with a significant increase in the number of γ-H2AX foci when compared with the control group (Figure 4C; *p* < 0.0001). We next performed a Comet assay to identify the DNA damage. This is a rapid and sensitive method that measures DNA damage in individual cells by alterations in the pattern of cellular elution through agarose gel showing in the form of an olive tail moment [40]. The values of the Olive tail moment in IL-24-expressing cells were higher than in the control group (Figure 4D; *p* < 0.0001), representing greater DNA damage. These findings suggest that IL-24 induces DNA damage.

### 2.5. IL-24 Triggers Apoptosis in H1299 Lung Cancer Cells

Studies have shown that HH signaling regulates the survival and proliferation of various cancer cells through GLI1 [11]. GLI1 plays a significant role in tumor growth, differentiation, metastasis, and therapy resistance 21. Tumor cells undergo apoptosis from various stimuli, including DNA-damaging agents, such as ionizing radiation and chemotherapeutic drugs. Genotoxic agents activate membrane death receptors, and the endogenous mitochondrial damage pathway induces apoptosis [43,44]. Pre-clinical and clinical studies have demonstrated that inhibition of HH signaling components can induce apoptosis either through activation of Fas signaling by increasing Fas, cleaved Caspase-3, and cleaved PARP, or by decreasing anti-apoptotic protein levels of the BCL-2 [40,41,45,46]. BCL-2 is the key direct transcriptional target of both GLI1 and GLI2 [11]. In addition, it has been reported that GLI1 and GLI2 modulate NSCLC proliferation by directly binding to the consensus GLI DNA binding sequence in cyclin D1 [47,48].

**Figure 5:**
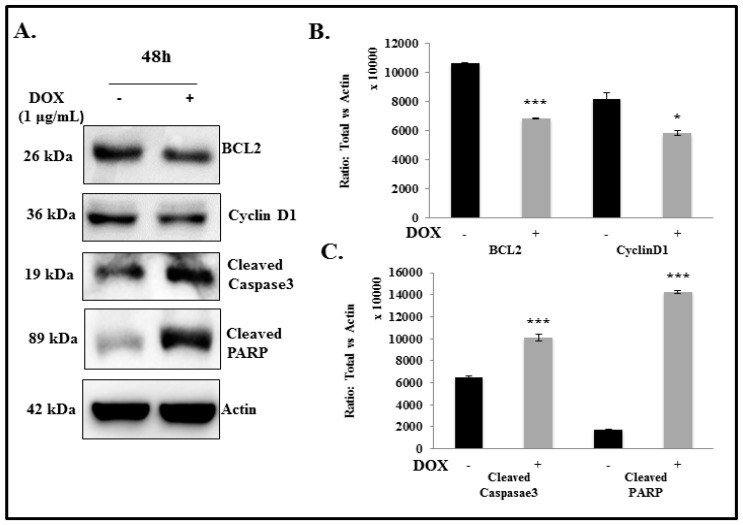
Effect of IL-24 on anti-apoptotic and apoptotic markers in H1299-IL24 lung cancer cells. (**A**) Western blotting analysis showed that expression of IL-24 protein in H1299-IL24 cells reduced the expression of BCL-2 and cyclin D1 and increased cleaved caspase3 and cleaved PARP expression at 48 h after doxycycline treatment. Beta-actin was used as a protein loading control. (**B**,**C**) Differences in the expression of the proteins were determined by semi-quantitative analysis and represented in graphical format. The experiment was performed at least two times. * Represents *p* < 0.05, *** represents *p* < 0.0001, ns = not significant. Bars denote standard deviation (SD).

In the present study, we found that IL-24 inhibits GLI1 expression and induces DNA damage in the lung cancer cells. We next raised the question of whether IL-24-mediated DNA damage involves the induction of cancer cell apoptosis. Recent data from our group, as well as from others, have shown that exogenous IL-24 expression induces tumor cell killing and cell cycle arrest, leading to apoptosis in cancer cells [28,37,49]. Consistent with these findings, we found that marked suppression of GLI1 was accompanied by a concomitant decrease in BCL-2 at 48 h (*p* < 0.0001) and Cyclin D1 (*p* < 0.05) expression at 48 and 72 h compared with untreated controls (Figure 5; Supplementary Figure S7). We also observed a significant increase in cleaved caspase-3 and cleaved PARP in cells induced with IL-24 at 48 and 72 h (Figure 5; Supplementary Figure S7; *p* < 0.0001). These findings suggest that IL-24-mediated GLI1 suppression triggers apoptosis by decreasing BCL-2 and Cyclin D1 expression and increasing cleaved Caspase-3 and cleaved PARP, suggesting increased apoptotic cell death, in addition to impaired cell proliferation.

### 2.6. IL-24 Induces DNA Damage and Apoptosis via GLI1 Inhibition

**Figure 6:**
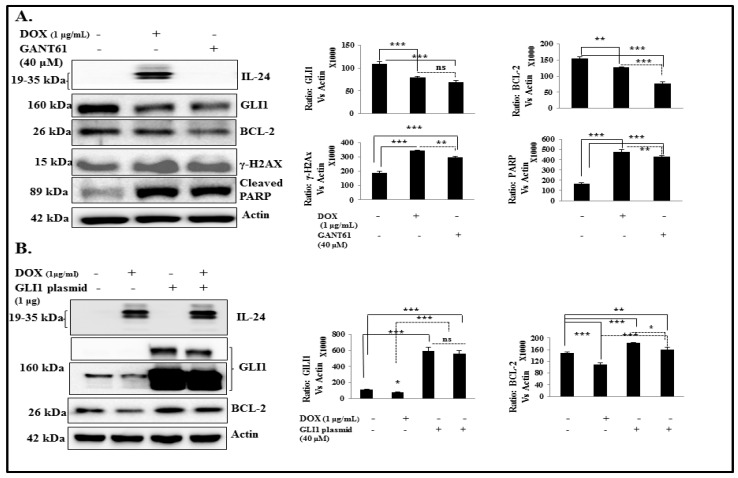
IL-24 induces DNA damage and apoptosis by suppressing GLI1. (**A**) H1299-IL-24 cells were either treated with GANT61 or induced with IL-24. Then, cells were subjected to molecular analysis at 48 h after DOX treatment. (**A**) Western blotting showed that GLI1 and its target BCL-2 expression were markedly reduced in IL-24-expressing cells and cells treated with GANT61 when compared with control cells. Increased γ-H2AX and cleaved PARP expression was observed in cells induced with IL-24, as well as in GANT61 treatment. Differences in the expression of the proteins were determined by semi-quantitative analysis and represented in graphical format. (**B**) H1299- IL-24 cells were untransfected or transfected with GLI1 plasmid, followed by treatment with or without 1 μg/mL doxycycline. Cell lysates were subjected to western blot analysis. GLI1-overexpressing cells showed an increase in BCL-2 expression compared with vector controls. However, upon induction of IL-24, GLI1-overexpressing cells showed a slight decrease in GLI1 with a significant decrease in BCL-2 expression. Beta-actin was used as a protein loading control. Differences in the expression of the proteins were determined by semi-quantitative analysis and represented in graphical format. Each experiment was performed at least two times. * Represents *p* < 0.05, ** represents *p* < 0.001, *** represents *p* < 0.0001, ns = not significant. Bars denote standard deviation (SD).

To evaluate whether IL-24 mediates DNA damage and apoptosis through inhibition of GLI1, we inhibited GLI1 expression using GANT61 in H1299-IL24 cells. Cells expressing IL-24 or treated with GANT61 showed a marked decrease in GLI1 (*p* < 0.0001) and BCL2 (*p* < 0.001), and increases in γ-H2AX (*p* < 0.0001) and cleaved PARP (*p* < 0.0001) expression when compared with controls (Figure 6A). Our data clearly indicate that IL-24 induces DNA damage and apoptosis through GLI1.

We next investigated whether IL-24 displays its inhibitory effect on GLI1 and its downstream targets in lung cancer cells overexpressing GLI1. H1299-IL-24 cells were transfected with GLI1 plasmid to accomplish GLI1 overexpression. We noted a significant increase in expression of GLI1 and its downstream target BCL-2 in cells overexpressing GLI1 plasmid, compared with vector controls. Upon induction of IL-24, GLI1-overexpressing cells showed a marginal decrease in GLI1 with a measurable decrease in BCL-2 (*p* < 0.05) expression when compared with GLI-overexpressing cells without IL-24 induction. These results suggest the existence of IL-24-mediated inhibitory activity in controlling GLI1 and its downstream signaling for the effective therapeutic outcome (Figure 6B).

Finally, we determined whether GANT61 treatment reduced pATM and pCHK2 akin IL-24 treatment shown in Figure 3. We observed GANT61 treated cells had marked reduction in pATM and pCHK2 protein expression. In fact, reduction in pATM expression was greater in GANT61 treated cells than in IL-24 expressing cells. These data indicate both IL-24 and GANT61 likely operate in a similar manner downstream of GLI1 (Supplementary Figure S8).

### 2.7. IL-24 Downregulates GLI1, Even under Treatment with Exogenous SHH Ligand

**Figure 7:**
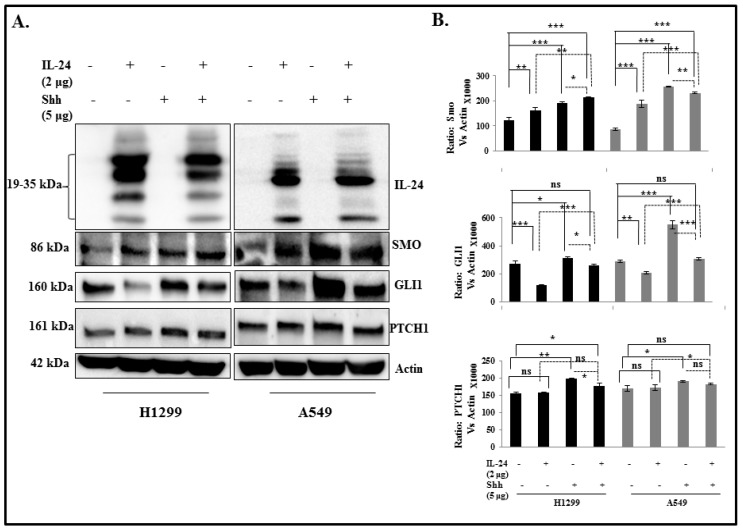
Effect of IL-24 on SHH signaling protein in the presence of exogenous Shh treatment. H1299 and A549 cells were treated with or without recombinant Shh (5000 ng/mL), transfected with or without IL-24 plasmid. (**A**) Western blot was performed to evaluate SMO, GLI1, and PTCH1 protein levels. Beta-actin was used as a protein loading control. (**B**) Differences in the expression of the proteins were determined by semi-quantitative analysis and represented in graphical format. The experiment was performed at least two times. * Represents *p* < 0.05, ** represents *p* < 0.001, *** represents *p* < 0.0001, ns = not significant. Bars denote standard deviation (SD).

To assess the inhibitory effect of IL-24 on SHH signaling proteins in the presence of exogenous SHH, NSCLC cells were treated with or without exogenous SHH/IL-24 and a combination of SHH and IL-24. It has been reported that NSCLC cells do not respond to exogenous SHH, as evidenced by no changes in either cell number or cell survival upon exposure to SHH. In accordance with these results, we observed no change in the SMO and PTCH1 protein expression between the groups (Figure 7). There was no influence of SHH treatment on cell number Thus, the saturated level of endogenous SHH does not permit NSCLC cells to respond to additional exogenous SHH to activate this pathway, suggesting that overexpression of SHH may be partly responsible for activation of the HH pathway in NSCLC cells. However, IL-24 induction decreased the expression of GLI1 even under treatment with exogenous SHH ligand in both H1299 and A549 cell lines (Figure 7). Indeed, the combination of IL-24 with SHH treatment showed a significant decrease in GLI1 expression when compared with SHH treatment alone. This finding clearly indicates that even in the presence of saturated SHH level in the NSCLC cells, expression of IL-24 only inhibits at the level of GLI1 expression, without affecting other components of the SHH pathway.

## 3. Discussion

Lung cancer-related death is primarily due to complexity in the molecular biology of the disease, resulting in drug resistance and metastasis [6]. Therefore, it is imperative to understand the molecular signaling involved in lung carcinogenesis and to identify new therapeutic targets. Studies have demonstrated that overexpression and activation of SHH signaling is involved in the development of many tumors, including lung cancer [9,10]. GLI1 is the major product of SHH signaling activation [11]. GLI1 can be activated non-classically by other pathways, such as PI3K-AKT, CXCR4, RAS, and TGF-beta, in tumor cells, independent of SHH1 [1,24]. Hence, inhibition at the level of GLI1 will halt the endpoint execution towards cancer cell survival and proliferation, making GLI1 a promising target for lung cancer treatment. However, the HH/GLI1-targeted drugs developed to date have shown poor efficacy in clinical studies due to resistance, non-specific toxicity, and lack of efficacy toward many tumors. Therefore, development and testing of new and effective drugs for lung cancer treatment is warranted.

In the present study, we tested the efficacy of IL-24 on GLI1 inhibition and observed IL-24 significantly reduced GLI1 protein expression in human lung tumor cell lines. Comparative analysis between stably and transiently transfected IL-24 in cancer cells revealed similar inhibitory effects on GLI1 expression. This finding shows that IL-24 expression is critically involved in the downregulation of GLI1 expression in lung cancer cells. Furthermore, IL-24 reduced GLI1 mRNA level post-transcriptionally, and thus modulated protein expression. The inhibitory activity on GLI1 was selective in tumor cells but not in normal cells. The underlying differences for tumor selectivity are unknown and not investigated in the present study.

The genomic stability of normal cells is maintained by the DDR signaling network, which consists of highly interconnected pathways and elicits a variety of cellular responses, including DNA repair, cell cycle arrest, and apoptosis [38]. Studies have shown that aberrant mutations in cancer cells deregulate the DDR machinery or DNA repair factors (either loss or gain), promoting the accumulation of DNA errors, genomic instability, survival, and resistance to DNA-damaging anti-cancer treatments [50]. One of the central members of the DDR machinery is the serine/threonine- protein kinase, ATM (ataxia–telangiectasia mutated). Studies have shown that hyperactivation of ATM promotes tumor progression, metastasis, and drug resistance [51,52,53]. Furthermore, the MRE11-RAD50-NBS1 (MRN) complex involved in the recruitment of ATM to sites of DNA double-strand breaks, and increased expression of MRN complexes have been observed in cancer cells. Hence, ATM and the MRN complex are the major sensors or mediators in the DDR and have been considered promising targets for sensitizing cancer cells to radiation or chemotherapy [50,54]; inhibiting ATM sensitized cancer cells and impaired cell migration and invasion in vitro [50,53,55,56]. Since aberrant activation of GLI1 influences DDR and repair signals and contributes to genomic instability [26], we tested whether IL-24-mediated GLI1 suppression impacted DDR machinery. Our data showed IL-24 reduced the expression of DDR proteins, ATM, RAD50, and MRE11 resulting in DNA damage as evidenced by the increase in γH2AX foci and Olive-tail moment. However, it is to be noted that while the changes in RAD50 and MRE11 expression were less than ATM, the biological significance brought by the subtle changes in the two proteins (RAD50 and MRE11) was not determined in this study. Nevertheless, the consequence of inhibiting DDR and GLI1 suppression converges in apoptotic cell death as evidenced by the reduction in cyclin D1 and Bcl-2 expression and an increase in caspase-3 and PARP cleavage [28,37,57]. Finally, the use of GLI inhibitor GANT61 and GLI1 overexpression plasmid DNA demonstrated that IL-24 specifically inhibits GLI1 and induces DNA damage.

**Figure 8:**
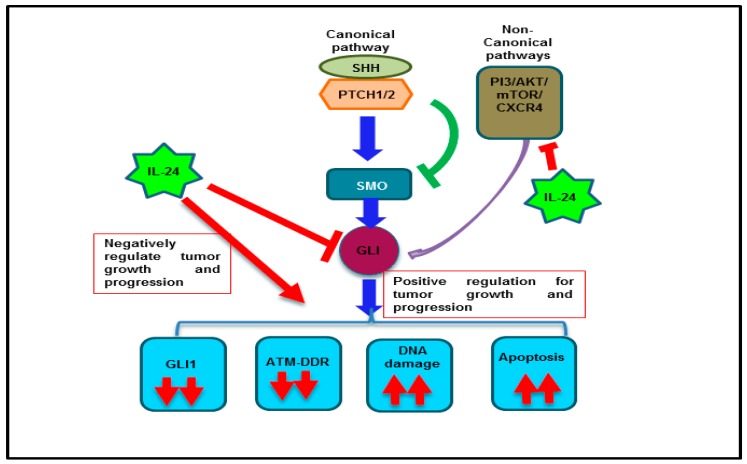
Schema showing the IL-24 regulation on GLI1 and its associated signaling in lung cancer cells.

Based on the observations made in the present study in conjunction with prior reports from our laboratory [35], we speculate that IL-24 directly regulates GLI1 and indirectly via the non-canonical pathway (Figure 8). In conclusion, our studies demonstrate IL-24 effectively suppresses GLI1 and offers a new IL-24-based treatment approach for targeting GLI1 and achieving improved treatment outcomes for lung cancer.

## 4. Materials and Methods

Human non-small cell lung cancer cell (NSCLC) lines (H1299, A549, HCC827, and H1975) and normal lung fibroblast cells (MRC-9 and WI38) (American Type Culture Collection (ATCC), Manassas, VA, USA) were cultured as previously described [35]. The cell lines were authenticated at the Genetic Resource Core Resource Facility, Johns Hopkins University, Baltimore, MD. In all experiments, untreated cells served as controls.

### 4.1. Stable Transfection of Inducible IL-24 Plasmid Vector in H1299 Cells

IL24-inducible plasmid and H1299-IL24 cell line used in the present study has been previously described [37].

### 4.2. Transient Transfection of IL-24 Plasmid

NSCLC cells (H1299 and A549) and normal lung fibroblast cells (MRC-9) were seeded in six-well tissue culture plates and were transiently transfected with 2 μg of plasmid expression vector carrying the IL-24 cDNA using DOTAP:cholesterol liposomes, as previously described [35]. After six hours of transfection, the tissue culture medium was aspirated and replenished with fresh medium. Untransfected cells served as controls. The cells were harvested at 48 h after transfection. Cell lysates were prepared and used for protein expression analysis.

### 4.3. Luciferase Reporter Assay

H1299-IL24 cells (1 × 10^5^) were seeded in six-well tissue culture plates, were transiently transfected with 100 ng of GLI1-Luc plasmid (Qiagen, Germantown, MD, USA), and were encapsulated in cationic DOTAP:cholesterol liposome [35,37]. After six hours of transfection, the tissue culture medium was removed and replenished with fresh medium supplemented with or without doxycycline (1 μg/mL). At 24 and 48 h after doxycycline treatment, the medium was removed and cells were washed gently with PBS. Cells were scraped and the supernatants were collected into 96-well white (opaque) plates (Corning, Tewksbury, MA, USA), cell lysates from each sample were transferred and 100 μL of luciferase assay reagent was added. Luciferase activity was measured with a PerkinElmer EnVision Multi label Reader (Waltham, MA, USA), according to the manufacturer’s instructions. For each sample, the results from triplicate wells were calculated and presented as the average of triplicate samples. Experiments were performed independently three times in order to determine statistical significance.

### 4.4. Exogenous SHH Treatment to NSCLC Cells

H1299 and A549 cells (1 × 10^5^) were seeded in six-well tissue culture plates, with 2 μg of a plasmid expression vector carrying the IL-24 cDNA using DOTAP:cholesterol liposomes, as previously described [35]. After six hours of transfection, the tissue culture medium was aspirated and replenished with fresh medium, with and without SHH (5 µg/mL). Untransfected cells served as controls. The cells were harvested at 24 h and cell lysates were prepared and used for protein expression analysis.

### 4.5. GLI1 Overexpression Studies

H1299-IL24 (1 × 10^5^) cells were seeded in six-well plates and transfected with 1 µg GLI1 DNA (Genecopeia, Rockville, MD, USA) using DOTAP:cholesterol liposome, as previously described [35,37]. Six hours after transfection, the medium was replaced with RPMI-1640 containing 2% tetracycline-free serum, with or without 1 μg/mL of doxycycline. Untransfected cells served as controls. After 48 h of incubation, the cells were harvested and total cell lysates were prepared for protein expression analysis.

### 4.6. Immunofluorescence Assay

H1299-IL24 cells were grown on coverslips placed in a six-well plate and treated with or without doxycycline (1 µg/mL). At 48 h after treatment, the cells were stained for γ-H2AX using anti-human γ-H2AX primary antibody (1:300; Cell Signaling Technology Inc., Danvers, MA, USA) and Alexa Fluor-488-labeled secondary antibody (1:300; Invitrogen, Thermo Fisher Scientific, Waltham, MA, USA) as previously described [58]. H2AX foci quantitated by counting a minimum of 50 nuclei per treatment.

### 4.7. Comet Assay

H1299-IL24 cells (0.2 × 10^6^/well) seeded in six-well plates were treated with or without doxycycline (1 µg/mL). The cells were harvested at 48 h and 72 h after treatment and subjected to Comet assay (Trevigen, Gaithersburg, MD, USA) [59]. The Olive tail moment was determined by screening 10 cells per field for five fields (50 cells in total) in each sample.

### 4.8. TCGA Lung Adenocarcinoma (LUAD) Data

The RNA-seq datasets from lung adenocarcinomas (LUAD) patients with included in the Cancer Genome Atlas (TCGA) dataset was downloaded using the UCSC cancer genome browser (https://genome-cancer.ucsc.edu/) and analyzed using Prism 7 software Version 7, GraphPad, San Diego, CA, USA).

### 4.9. Determination of GLI1 Expression in LUAD Pathological Stages

Patients GLI1 expression levels were segregated based on pathological stages (Stage I, VII, III, and IV) from the LUAD dataset downloaded from TCGA database. The box and whisker plot was constructed using Prism 7 software (Version 7, GraphPad, San Diego, CA, USA).

### 4.10. Survival Curve Analysis

Lung adenocarcinoma dataset was downloaded from GEO, EGA and TCGA databases using KMplot browser and analyzed for the correlation between overall survival (OS) and GLI1 expression (www.kmplot.com).

### 4.11. Real-Time PCR Analysis

Total RNA from the control and doxycycline-treated H1299-IL24 cells was isolated using Trizol (Life Technologies, Grand Island, NY, USA) and was subjected to reverse transcription using an iScript cDNA synthesis kit (Bio-Rad, Hercules, CA, USA). The complementary DNA (cDNA) was subsequently used to perform real-time (RT)-PCR (Bio-Rad CFX96 Touch Real-Time PCR Detection System, Hercules, CA, USA) with SYBR chemistry using iQTM SYBR Green super mix (Bio-Rad) and human GLI1-specific oligonucleotide primers (Forward-5′AGCTAGAGTCCAGAGGTTCAA 3′-Sense, Reverse-5′TAGACAGAGGTTGGGAGGTAAG 3′-Antisense), GLI2-specific oligonucleotide primers (Forward-5′GTTCATCGCCTTCCTGAGATA 3′-Sense, Reverse-TGGACGACTCACCTACAGTAT 3′-Antisense), (Integrated DNA Technologies, Coralville, IA, USA). Thermal cycling was programmed as follows: 95 °C for 30 s, followed by 40 cycles of 95 °C for 20 s, 62 °C for 20 s, and 72 °C for 20 s. The crossing threshold (Ct) value assessed by RT-PCR was noted for the transcripts and normalized with human 18S mRNA (Forward-5′-CAGCCACCCGAGATTGAGCA-3′ and Reverse-5′-TAGTAGGGACGGGCGGTGTG-3′; Integrated DNA Technologies). The changes in mRNA was expressed as fold changes relative to control ± the standard deviation (SD).

To determine the stability of GLI1 mRNA, cells (1 × 10^5^) were treated with or without doxycycline (1 μg/mL) for 48 h. The following day, the cells were treated with or without actinomycin D (3 μM; Amersco LLC, Solon, OH, USA) and were harvested at 30 min, 1 h, 2 h, 3 h, and 4 h. Total RNA prepared from the harvested cells was used to determine the GLI1 mRNA levels by RT-PCR, as described above. GLI1 mRNA half-lives were calculated from typical decay curves by linear regression between 0 h and 4 h [60]. Values ±SD are based on at least two independent experiments.

### 4.12. Western Blotting Analysis

Cells receiving various treatments and collected at various time points were subjected to western blot analysis, as previously described [35,37]. Primary antibodies against IL-24 (1:2000; Introgen Therapeutics, Houston, TX, USA), GLI1, GLI2, PTCH2, SMO, γ-H2AX, PARP, Capase3, pATM^S1981^, ATM, pCHK2^T68^, CHK2, MRE11 (1:1000; Cell Signaling Technology Inc.), PTCH1 (Abcam, Cambridge, MA, USA), RAD50 (Santa Cruz Biotechnology, Dallas, TX, USA), and beta-actin (1:2000; Sigma Chemicals, St. Louis, MO, USA) were purchased and used as recommended by the manufacturers. Proteins were detected using the appropriate secondary antibodies (Santa Cruz Biotechnology, Inc., and Jackson Immuno Research Laboratories, Inc., West Grove, PA, USA) and an enhanced chemiluminescence kit (Thermo Scientific). Protein levels were detected using a chemiluminescence imaging system (Syngene, Frederick, MD, USA) and quantified using GelQuant software (V1.7.8, University of California- San Francisco, CA, USA).

### 4.13. Statistical Analysis

Unless otherwise stated, all experiments were performed a minimum of two times and data are shown as mean ±standard deviation (SD). Two-sample *t*-tests were performed to detect the difference between a single group and the control. Univariate statistical significance was determined by one-way analysis of variance (ANOVA) with Dunnett or Tukey adjustments performed to compare multiple (three or more) group means with the control or to make all possible pairwise comparisons, respectively. SAS (v. 9.4, SAS, Cary, NC, USA) was used to conduct all analyses. Differences with *p* < 0.05 were deemed significant.

## 5. Conclusions

The present study demonstrates the ability of IL-24 to effectively suppress GLI1 in lung cancer cells and induce DNA damage leading to apoptotic cell death. Furthermore, IL-24 could effectively reduce SHH ligand-mediated GLI1 activation. The study results show IL-24 as an effective anticancer drug for treating lung cancer.

## Supplementary Materials

The following are available online at https://www.mdpi.com/2072-6694/11/12/1879/s1, Figure S1: IL-24 reduced GIL1 expression in H1299-IL24 lung cancer cells. Figure S2: Determination of IL24 inhibitory activity on GL12 expression in H1299-IL24 lung cancer cells. Figure S3: Inhibitory activityIL-24 on GLI1 expression in H1299 and A549 lung cancer cells. Figure S4: Assesment of IL-24 inhibitory on GLI1 expression in normal fibroblast cells. Figure S5: Effect of IL-24 on DDR protein expression in H1299 cells. Figure S6: IL-24 induces DNA damage. Figure S7: IL-24 induce apoptosis in H1299 cells. Figure S8: Comparision of IL-24 and GANT61 inhibitory activity on ATM and CHK2.
