# Supplementary material for: IL-24 Inhibits Lung Cancer Growth by Suppressing GLI1 and Inducing DNA Damage
Source: Cancers (Basel). 2019 Nov 27;11(12):1879. doi: 10.3390/cancers11121879 (PMC6966580; doi:10.3390/cancers11121879)
Supplement: Supplementary file 1 [file cancers-11-01879-s001.zip › cancers-604942-supplementary-final.pdf]

cancers

MDPI

Article

IL-24 Inhibits Lung Cancer Growth by Suppressing GLI1 and Inducing DNA Damage

Janani Panneerselvam [1,2], Akhil Srivastava [1,2], Meghna Mehta [2,3], Allshine Chen [2,4], Yan D. Zhao [2,4], Anupama Munshi [2,3], and Rajagopal Ramesh [1,2,5,*]

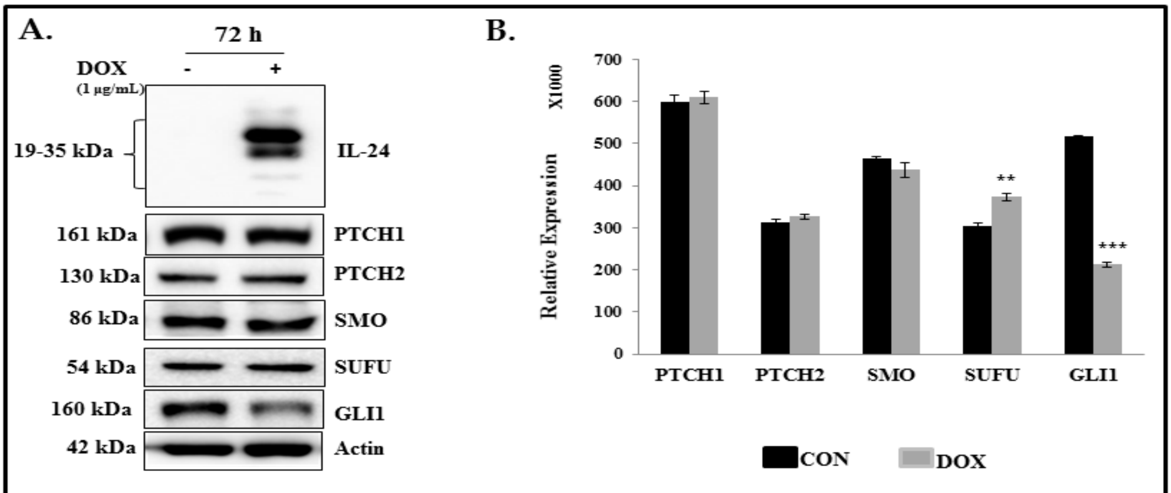

Figure S1. IL-24 reduced GLI1 expression in H1299-IL24 lung cancer cells. (A) IL-24 reduced GLI1 expression, but increased SUFU expression at 72 h in H1299-IL24 cells compared with control cells. No marked change in PTCH1, PTCH2 and SMO expression was observed IL-24 expressing cells compared with control cells. **(B)** Differences in the expression of the proteins were determined by semi-quantitative analysis and represented in graphical format. ** Represents $p < 0.001$, *** represents $p < 0.0001$. Bars denote standard deviation (SD).

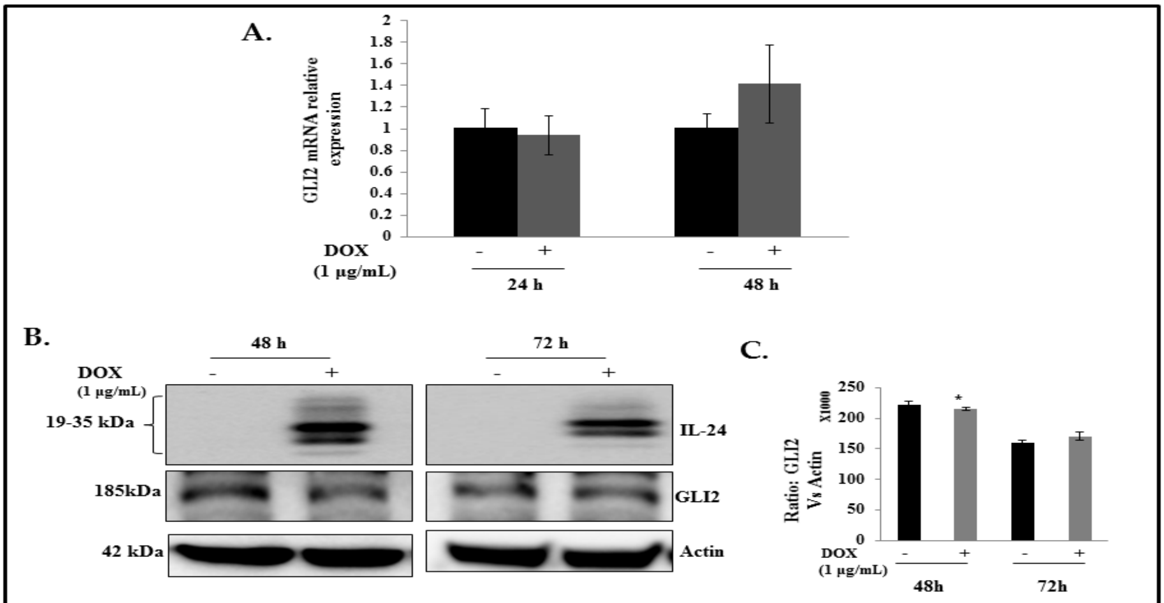

Figure S2. Determination of IL-24 inhibitory activity on GLI2 expression in H1299-IL24 lung cancer cells. IL-24 did not exhibit inhibitory activity on GLI2 **(A)** mRNA or **(B)** protein expression in H1299-IL24 cells. **(C)** Differences in the expression of the proteins were determined by semi-quantitative analysis and represented in graphical format. * Represents $p < 0.05$. Bars denote standard deviation (SD).

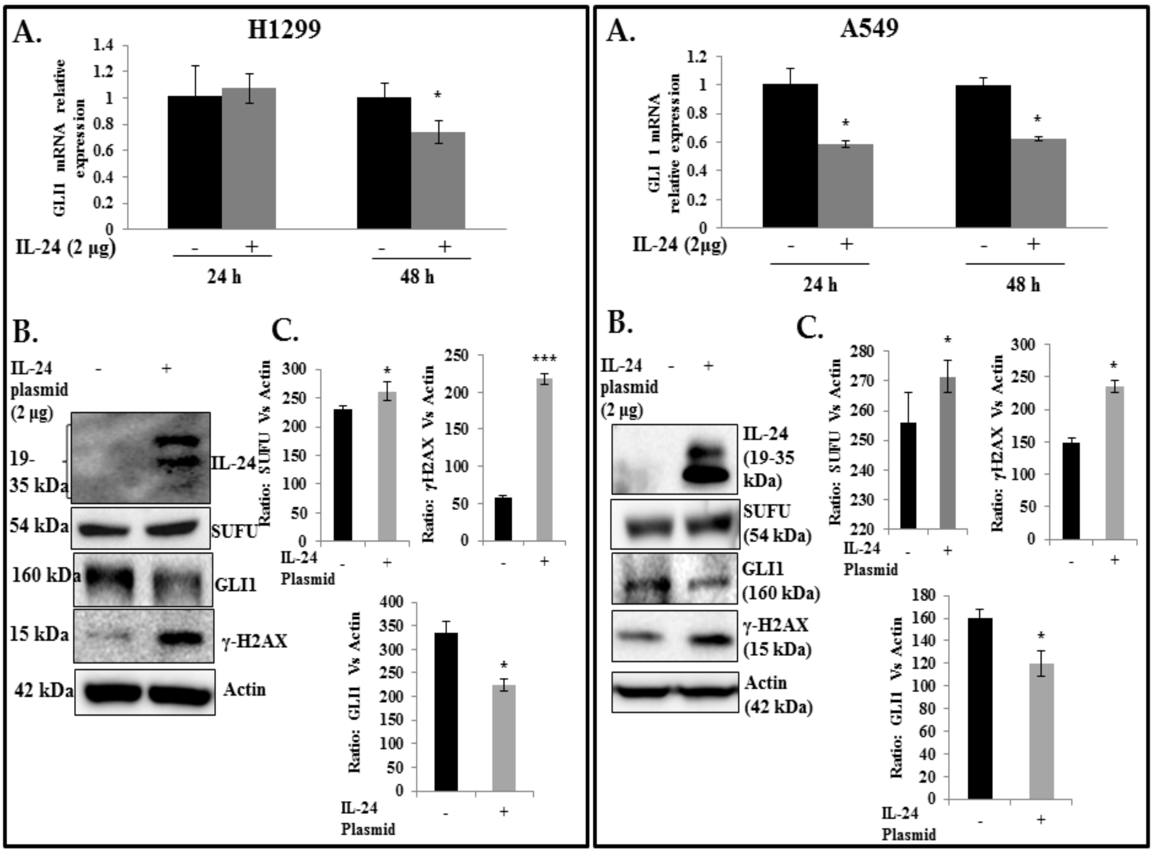

Figure S3. Inhibitory activity IL-24 on GLI1 expression in H1299 and A549 lung cancer cells. Transient transfection study showed IL-24 inhibited GLI1 and induced DNA damage in both H1299 and A549 cells at the two points tested. (**A**) GLI1 mRNA expression. (**B**) Western blotting for protein expression. (**C**) Differences in the expression of the proteins were determined by semi-quantitative analysis and represented in graphical format. * Represents p < 0.05, *** represents p < 0.0001. Bars denote standard deviation (SD).

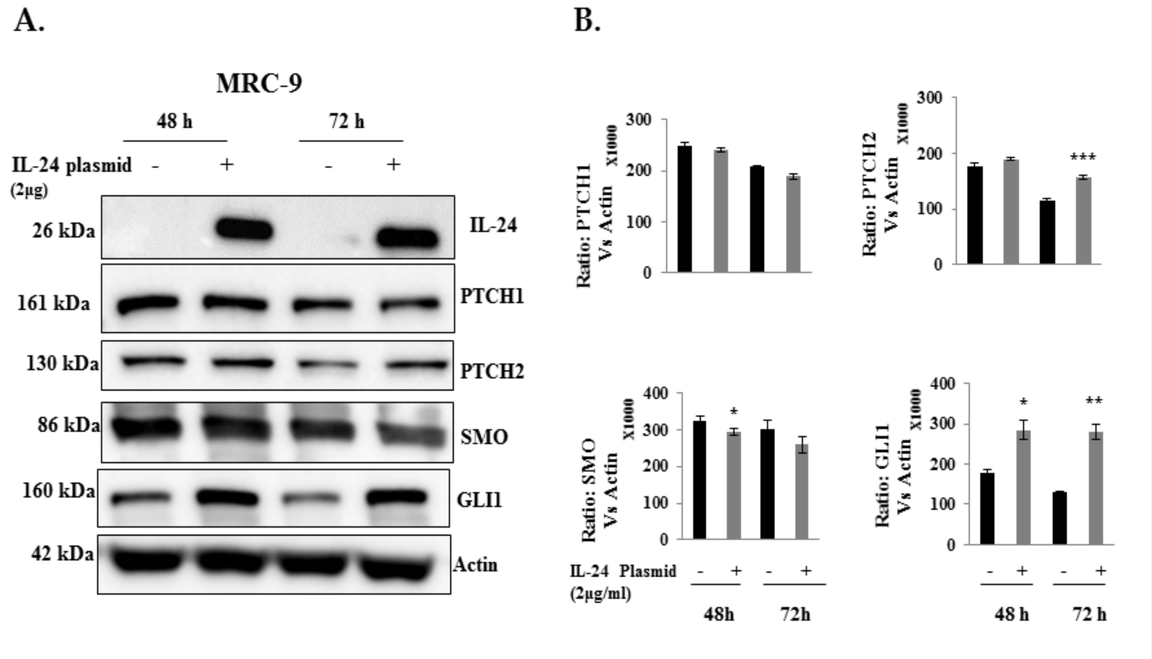

Figure S4. Assessment of IL-24 inhibitory activity on GLI1 expression in normal fibroblast cells. (**A**) Transient expression of IL-24 showed no inhibitory activity on GLI1, PTCH1 or PTCH2. However, reduction in SMO expression was observed. (**B**) Differences in the expression of the proteins were determined by semi-quantitative analysis and represented in graphical format. * Represents p < 0.05; ** represents p < 0.001; *** represents p < 0.0001. Bars denote standard deviation (SD).

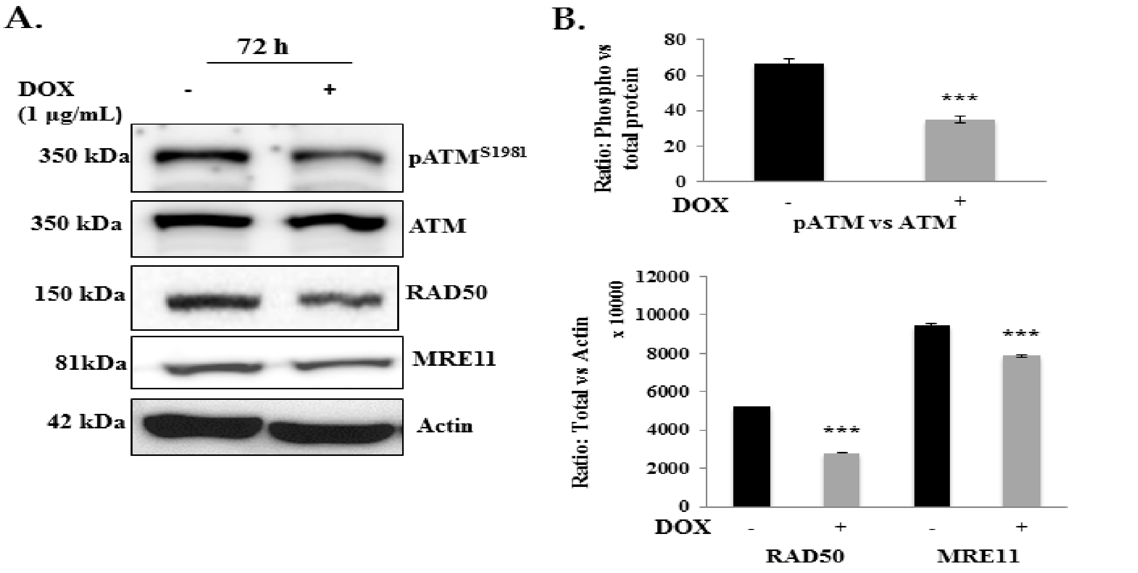

Figure S5. Effect of IL-24 on DDR protein expression in H1299 cells. (**A**) Induction of IL-24 protein expression in H1299-IL-24 cells reduced pATM^{S1981}, RAD50, and MRE11 at 72 h. (**B**) Differences in the expression of the proteins were determined by semi-quantitative analysis and represented in graphical format. ** Represents p < 0.001, *** represents p < 0.0001. Bars denote standard deviation (SD).

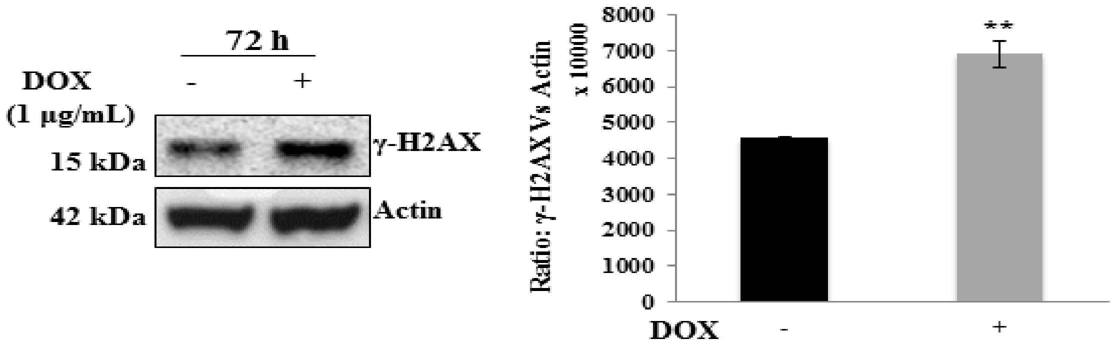

Figure S6. IL-24 induces DNA damage. Induction of IL-24 expression in H1299-IL-24 cells resulted in increased γ-H2AX expression indicating occurrence of DNA damage at 72 h. Differences in the expression of the proteins were determined by semi-quantitative analysis and represented in graphical format. ** Represents p < 0.001. Bars denote standard deviation (SD).

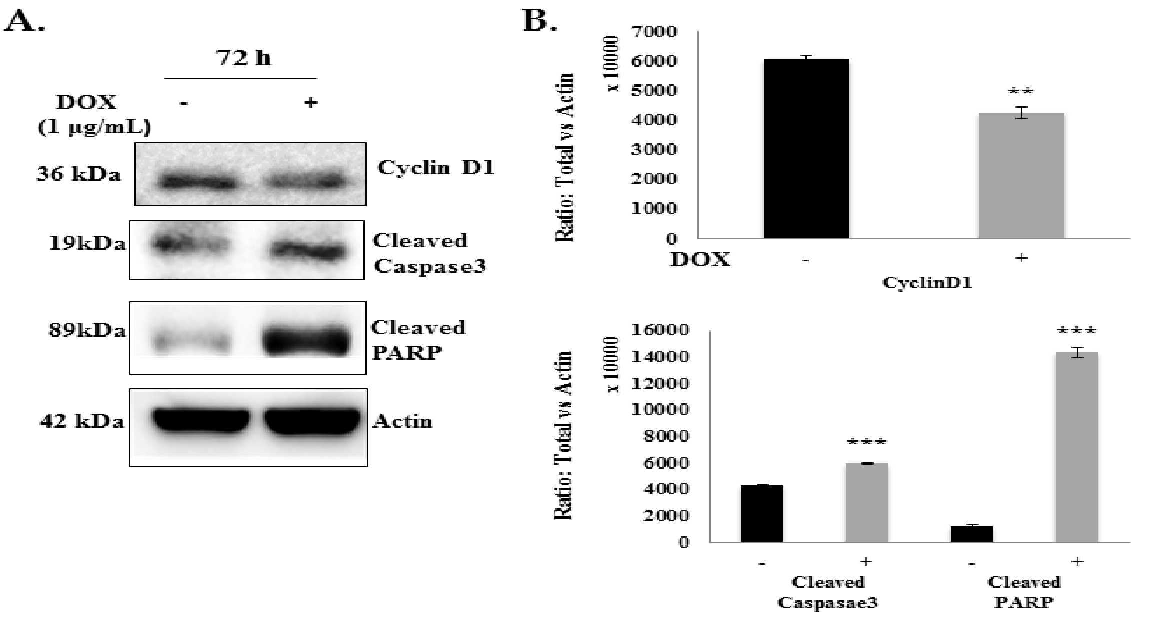

Figure S7. IL-24 induces apoptosis in H1299 cells. (A) Western blotting showed IL-24 expression reduced cyclin D1 and activated cleavage of caspase 3 and PARP. (B) Differences in the expression of the proteins were determined by semi-quantitative analysis and represented in graphical format. ** Represents $p < 0.001$, *** represents $p < 0.0001$. Bars denote standard deviation (SD).

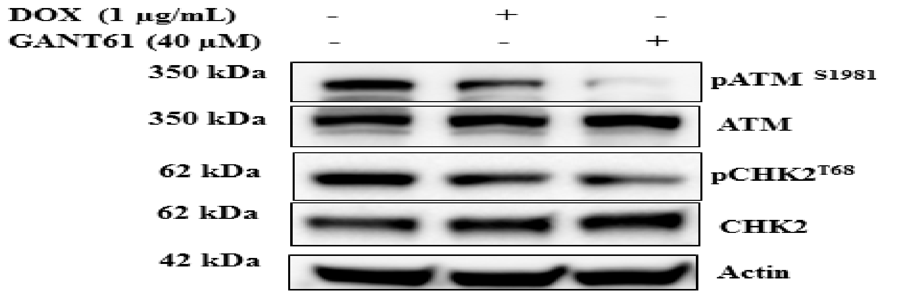

Figure S8. Comparison of IL-24 and GANT61 inhibitory activity on ATM and CHK2. Both, IL-24 and GANT61 reduced $pATM^{S1981}$ and $pCHK2^{T68}$ in H1299-IL-24 cells at 72 h. However, GANT61 exerted greater inhibitory activity on $pATM^{S1981}$ and $pCHK2^{T68}$.

© 2019 by the authors. Licensee MDPI, Basel, Switzerland. This article is an open access article distributed under the terms and conditions of the Creative Commons Attribution (CC BY) license (http://creativecommons.org/licenses/by/4.0/).
