# Supplementary material for: IL-24 Inhibits Lung Cancer Growth by Suppressing GLI1 and Inducing DNA Damage
Source: Cancers (Basel). 2019 Nov 27;11(12):1879. doi: 10.3390/cancers11121879 (PMC6966580; doi:10.3390/cancers11121879)
Supplement: Supplementary file 1 [file cancers-11-01879-s001.zip › western blot figures.pdf]

Figure 1 (tumor cells)

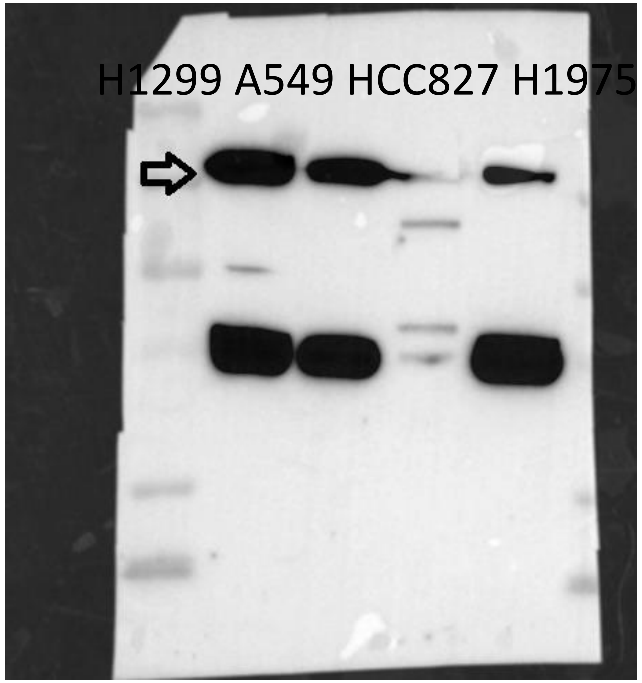

PTCH1

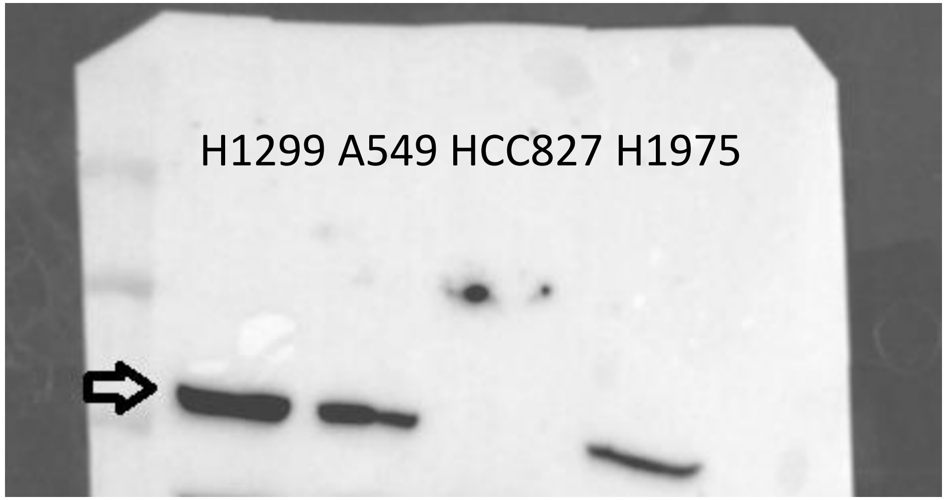

PTCH2

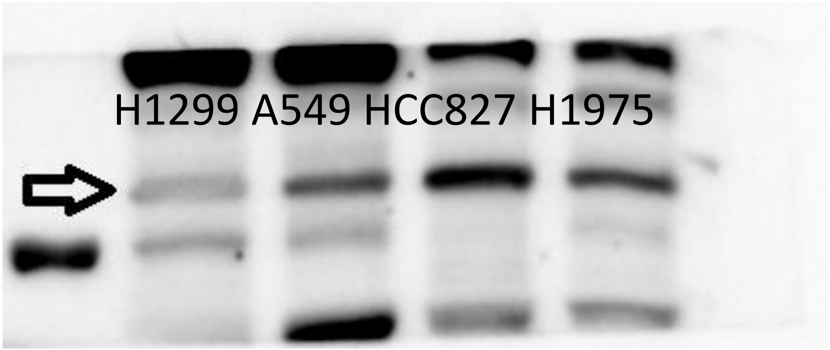

SMO

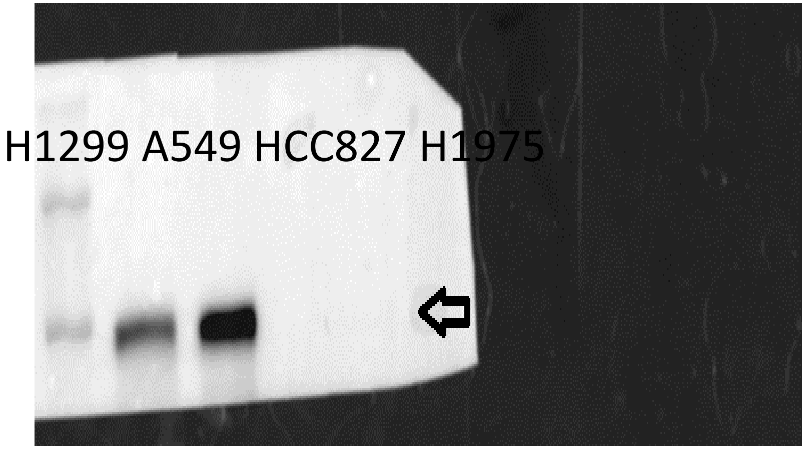

GLI1

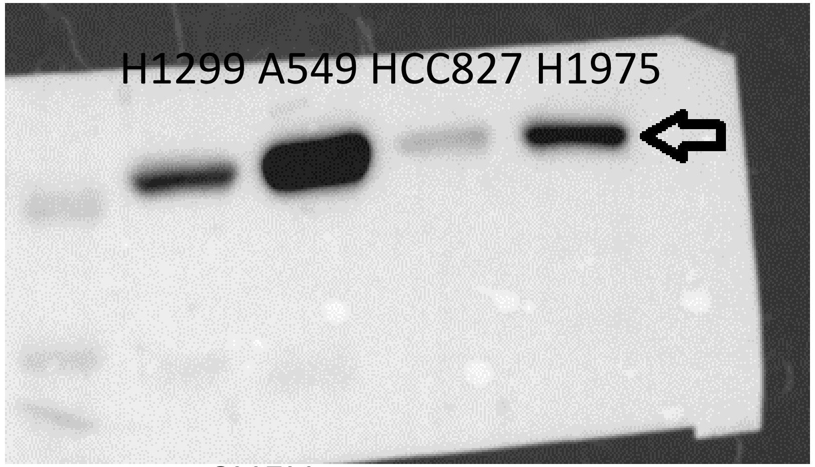

SUFU

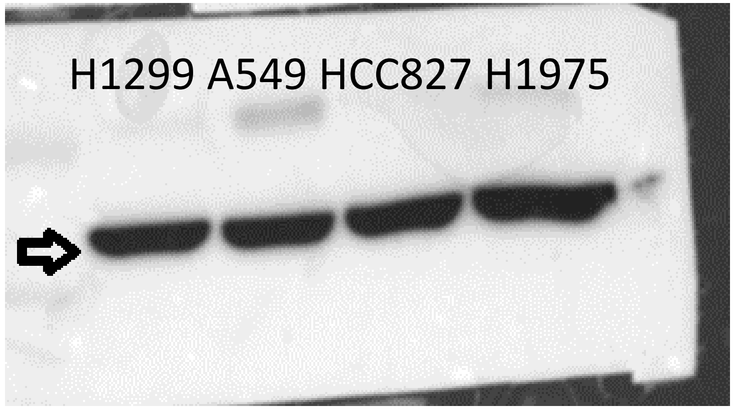

Actin

Figure 1 (Normal cells)

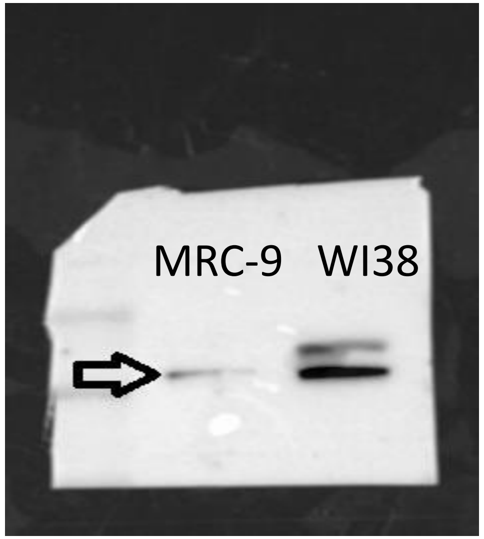

PTCH1

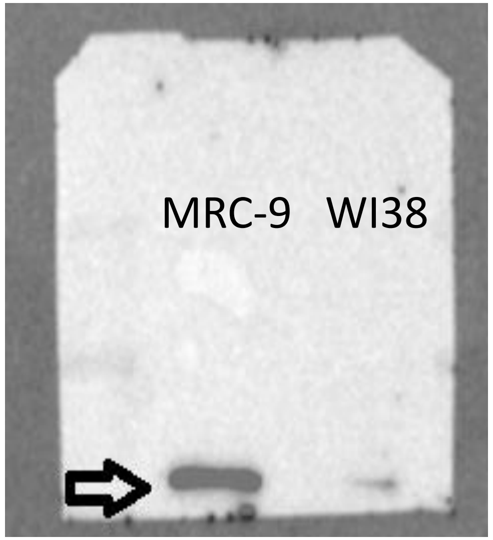

PTCH2

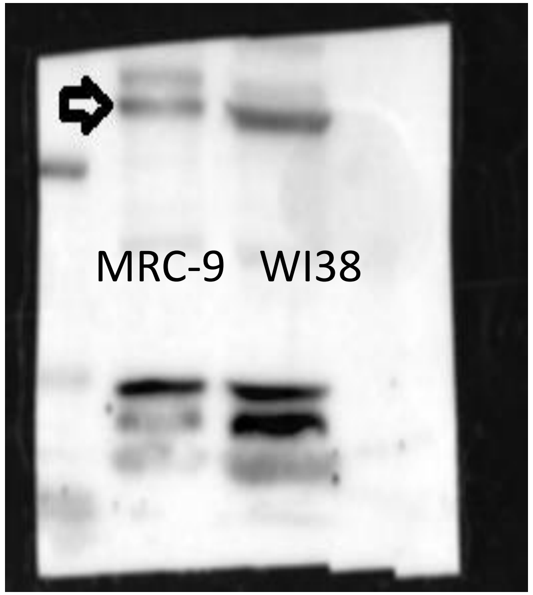

SMO

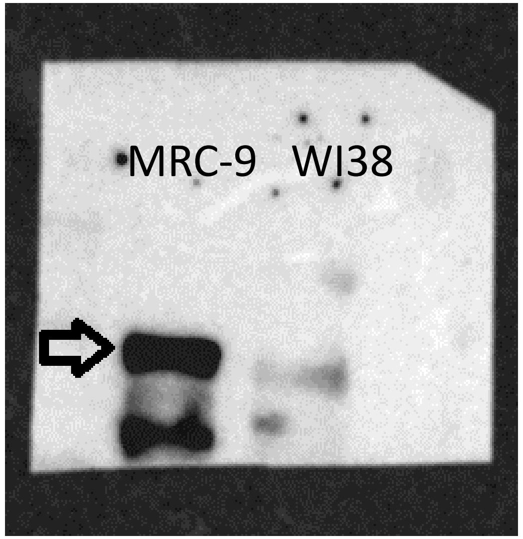

GLI1

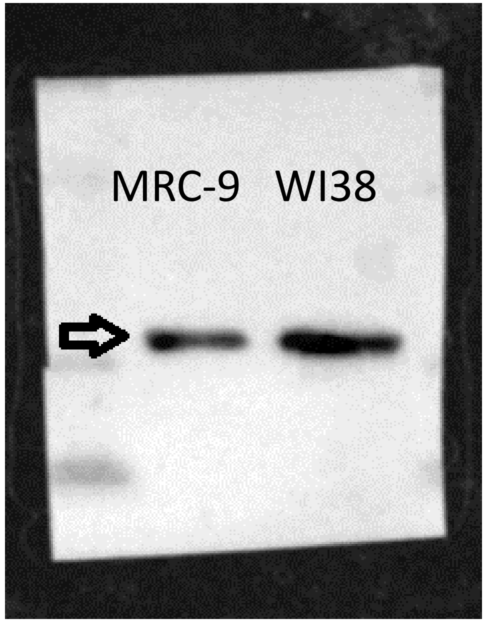

SUFU

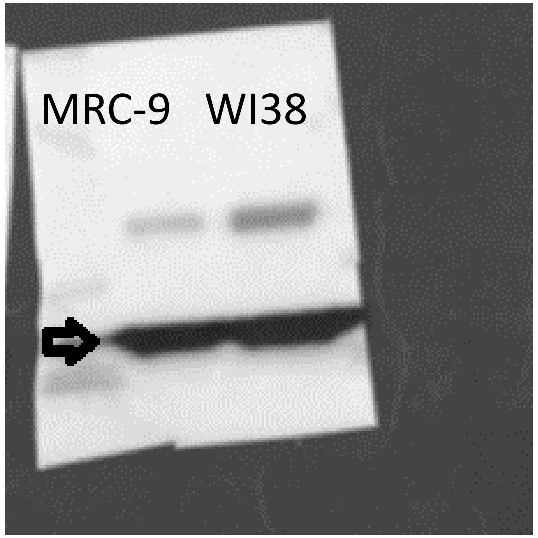

Actin

Figure 2 A

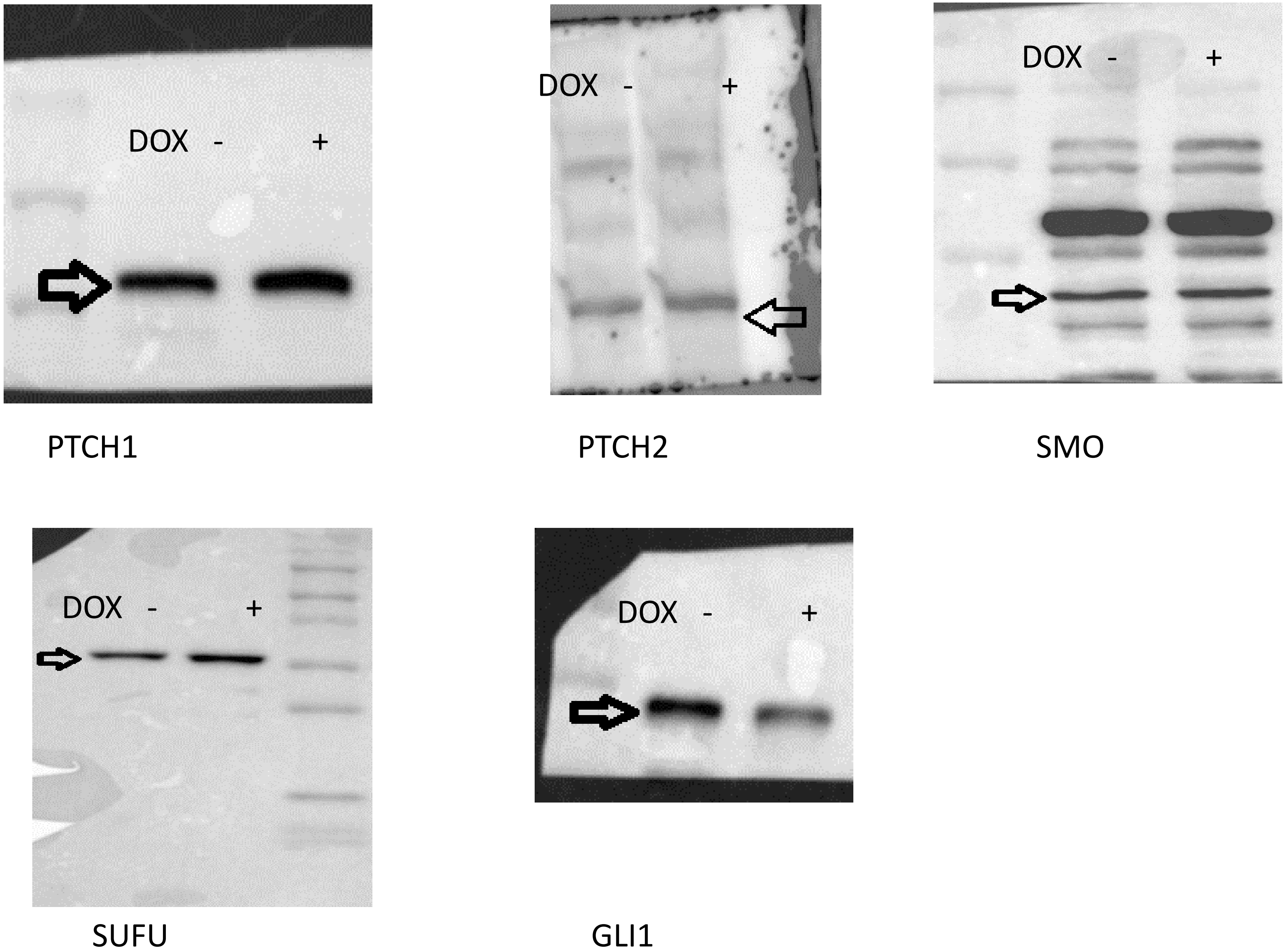

Figure 3

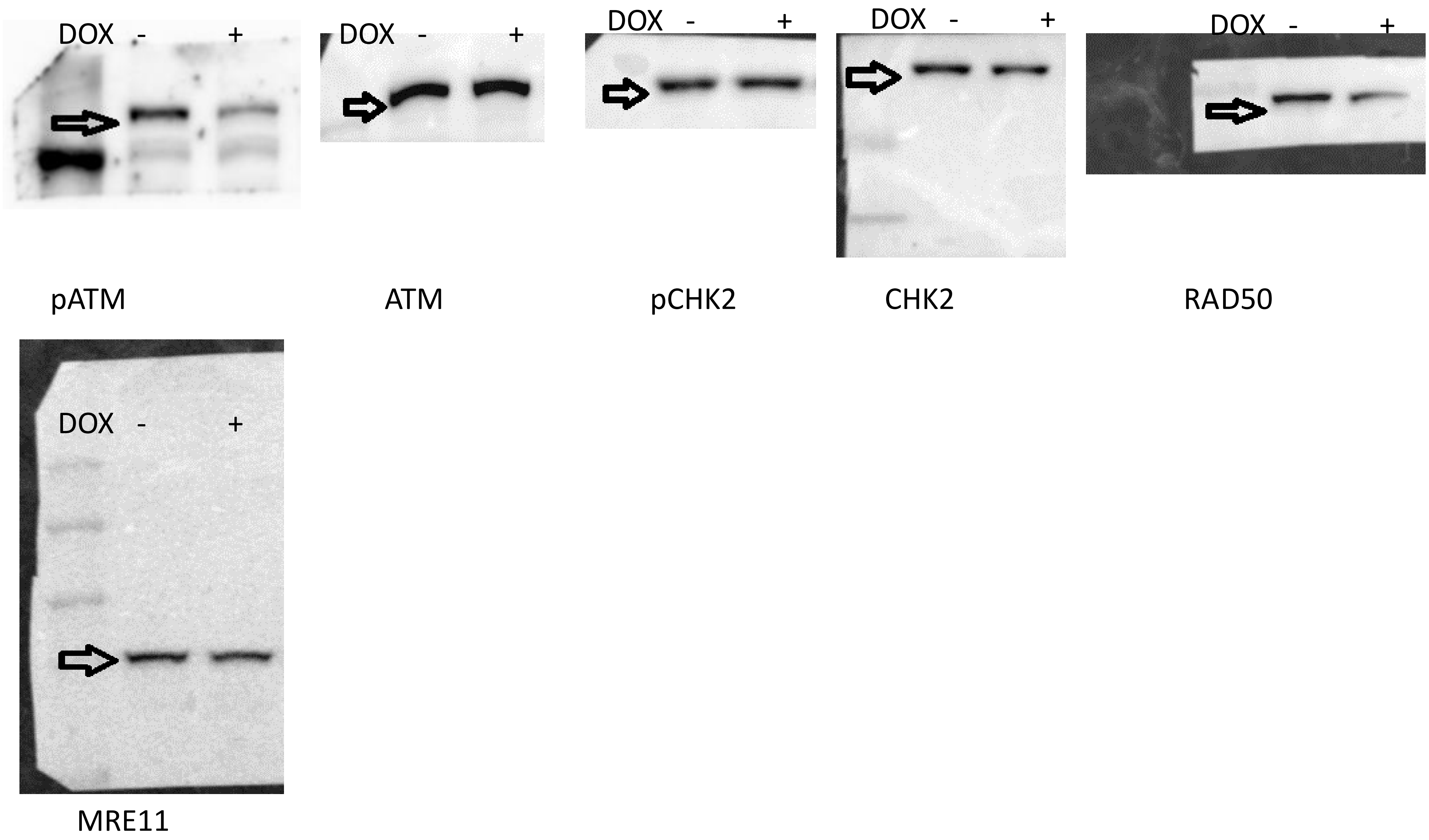

Figure 4

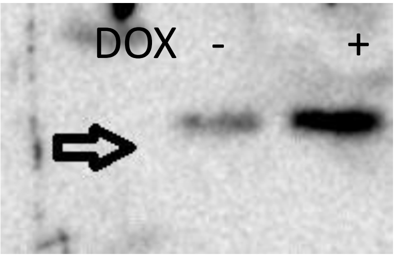

Gamma H2AX

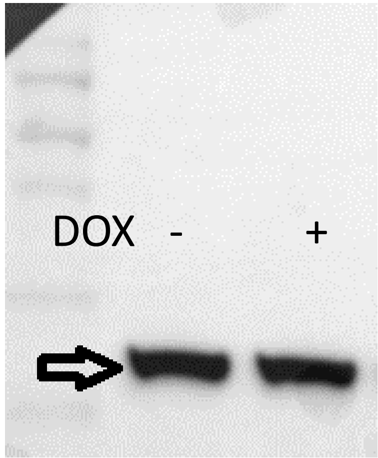

Actin

Figure 5

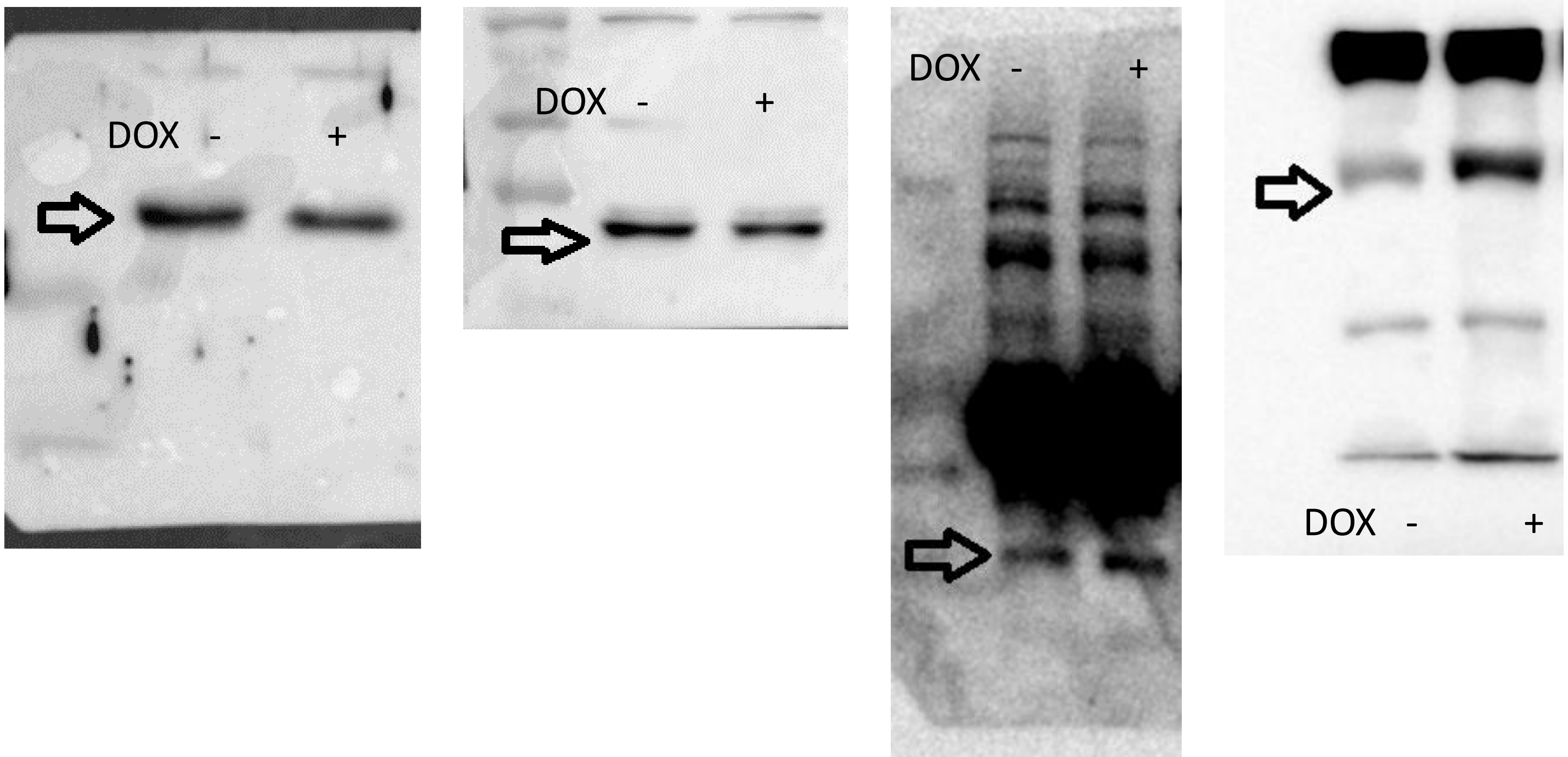

Bcl2 | Cyclin D1 | Cleaved Caspase 3 | Cleaved PARP

Figure 6A

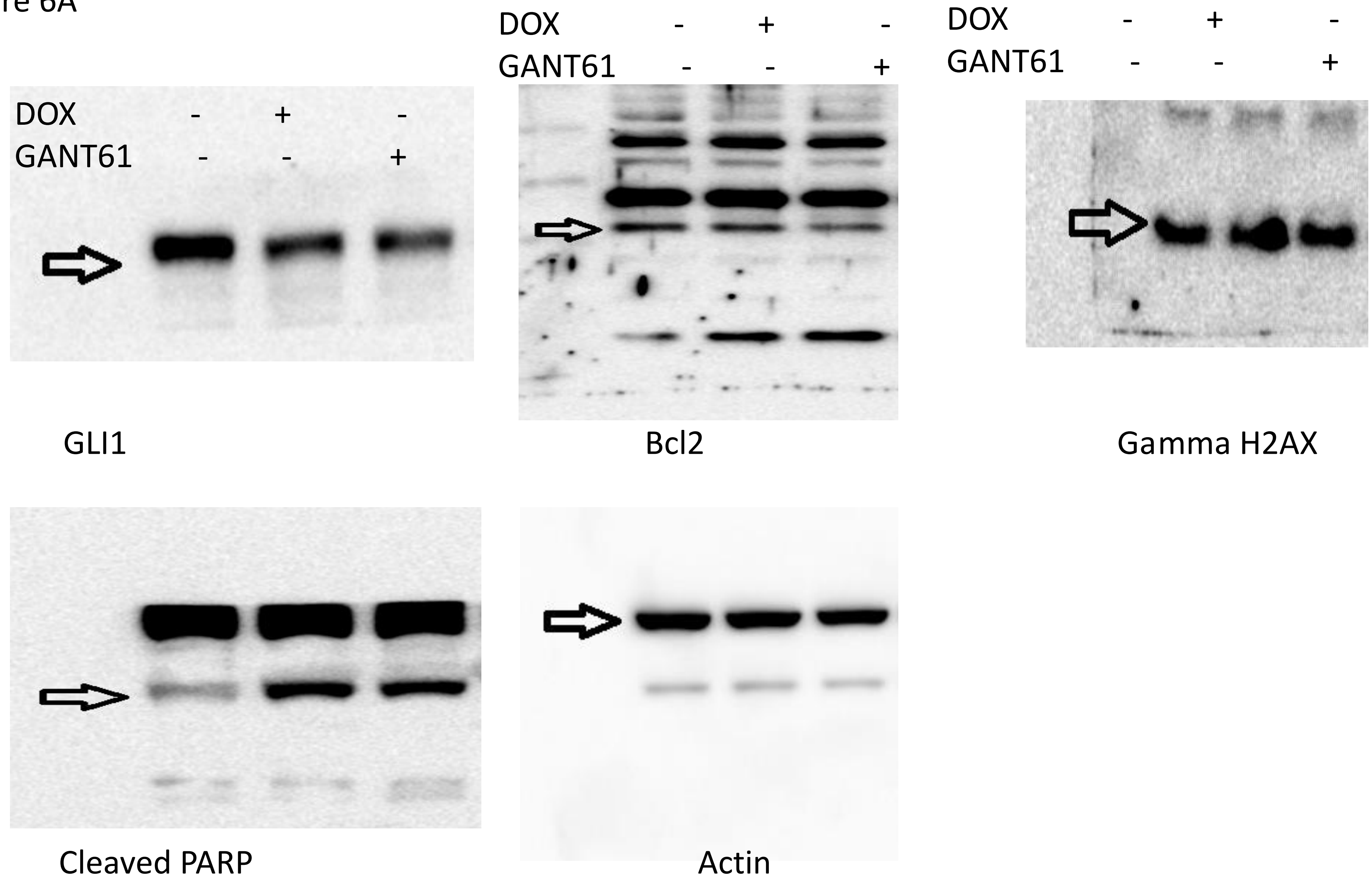
DOX - + -
GANT61 - - +
GLI1
DOX - + -
GANT61 - - +
Bcl2
DOX - + -
GANT61 - - +
Gamma H2AX
Cleaved PARP
Actin

Figure 6B

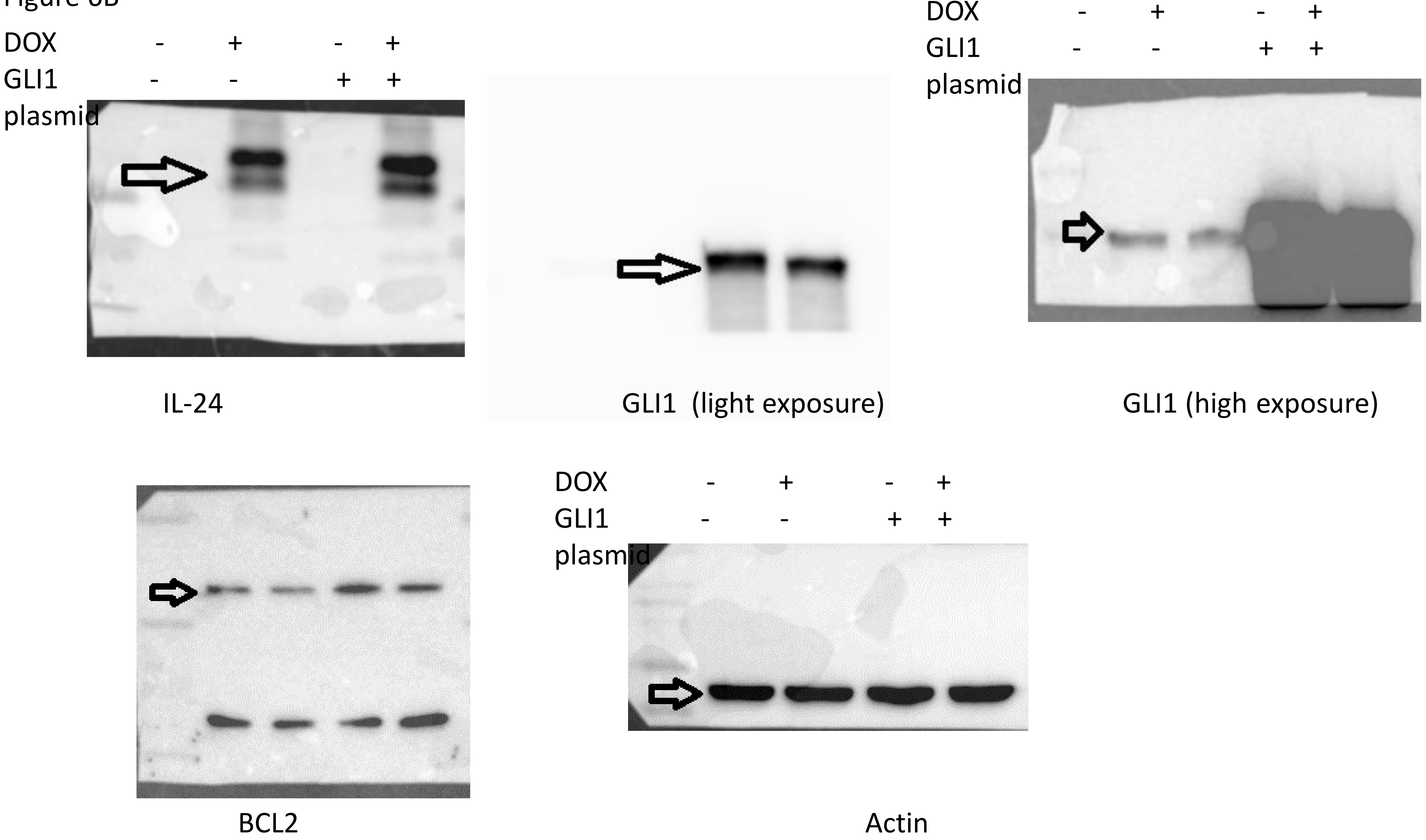
DOX - + - +
GLI1 plasmid - - + +
IL-24
GLI1 (light exposure)
DOX - + - +
GLI1 plasmid - - + +
GLI1 (high exposure)
BCL2
DOX - + - +
GLI1 plasmid - - + +
Actin

Figure 7 (H1299)

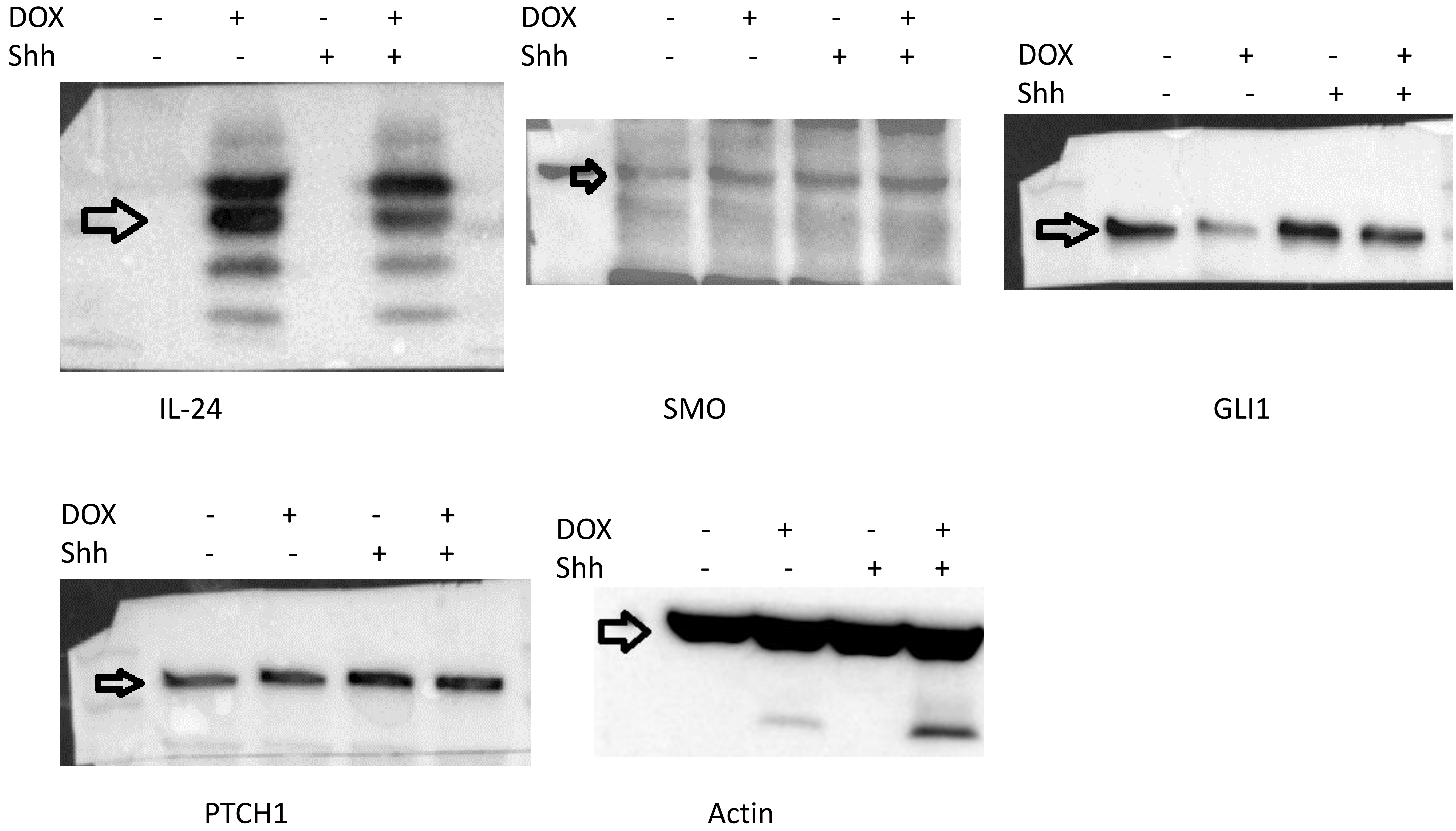

Figure 7 (A549)

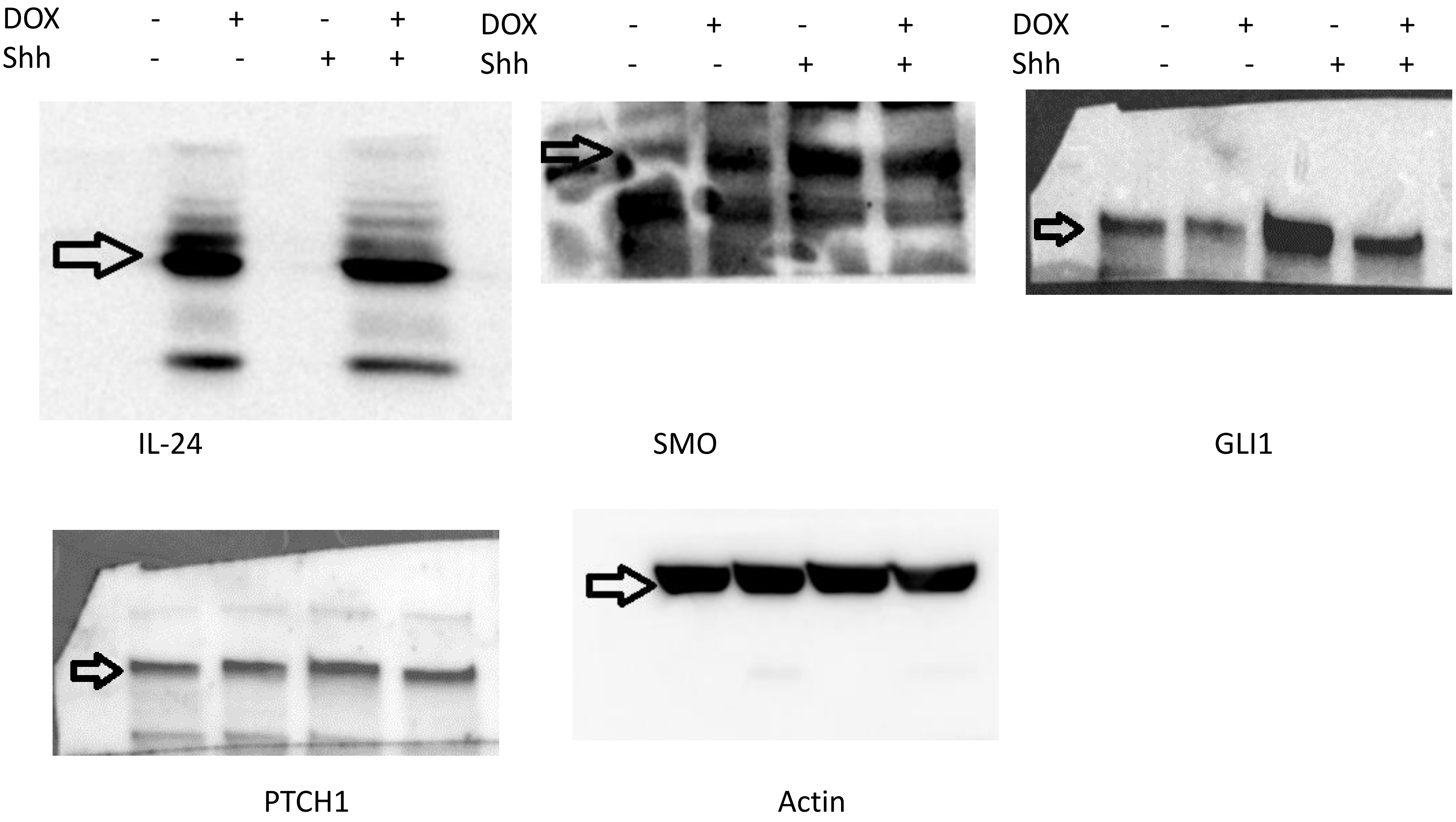

Figure S1

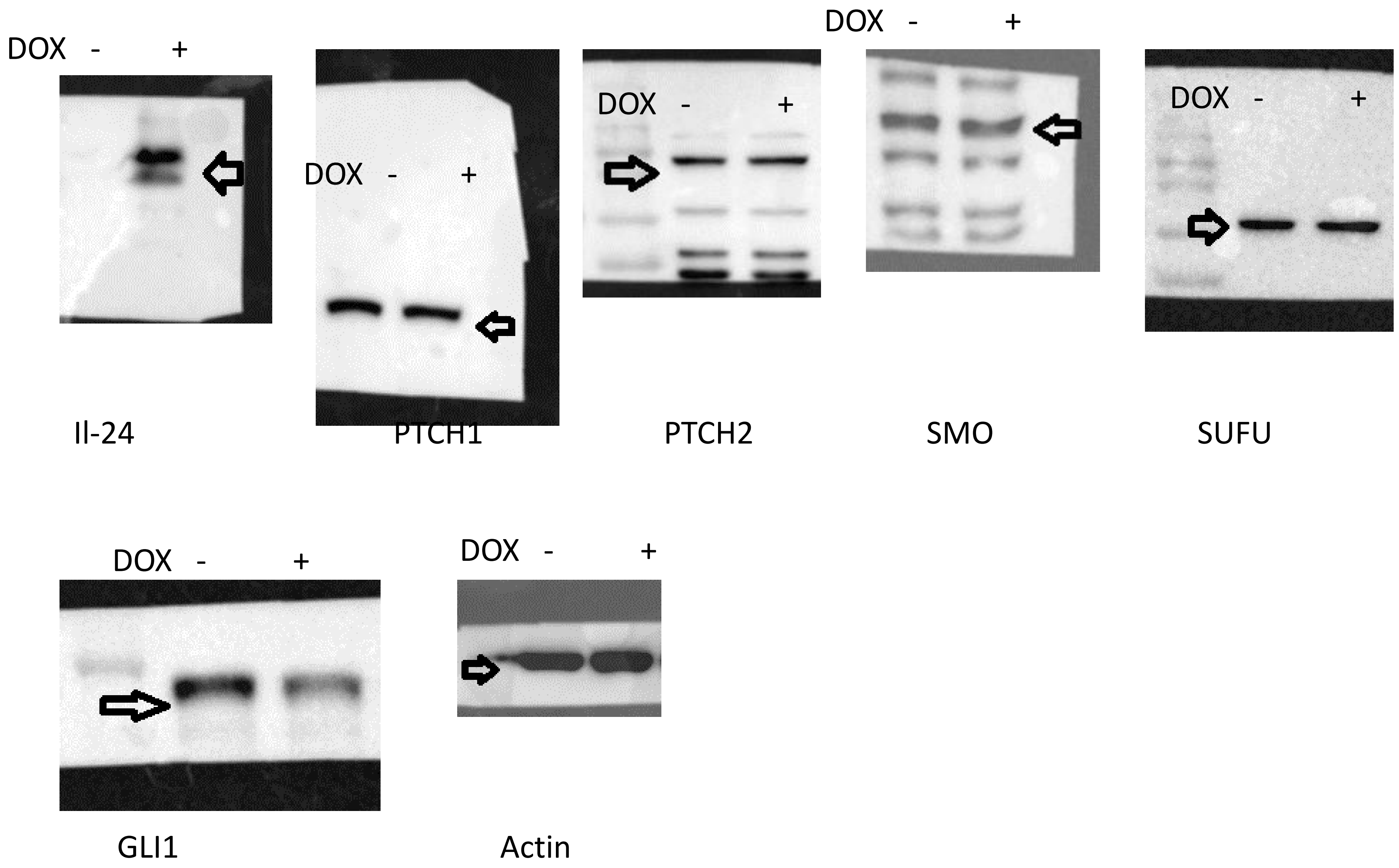
DOX - +
Il-24
DOX - +
PTCH1
DOX - +
PTCH2
DOX - +
SMO
DOX - +
SUFU
DOX - +
GLI1
DOX - +
Actin

Figure S2

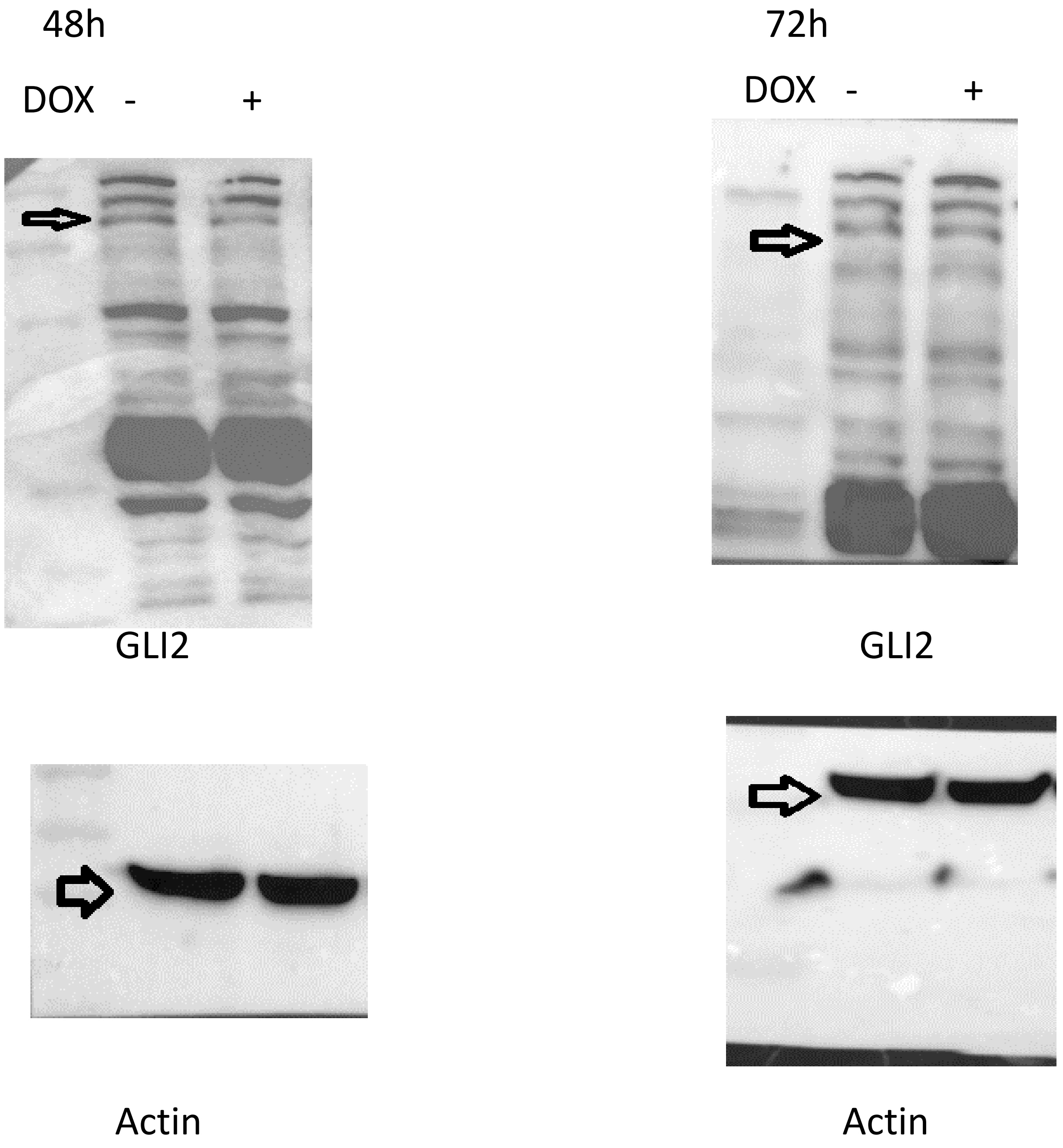

Figure S3 (H1299)

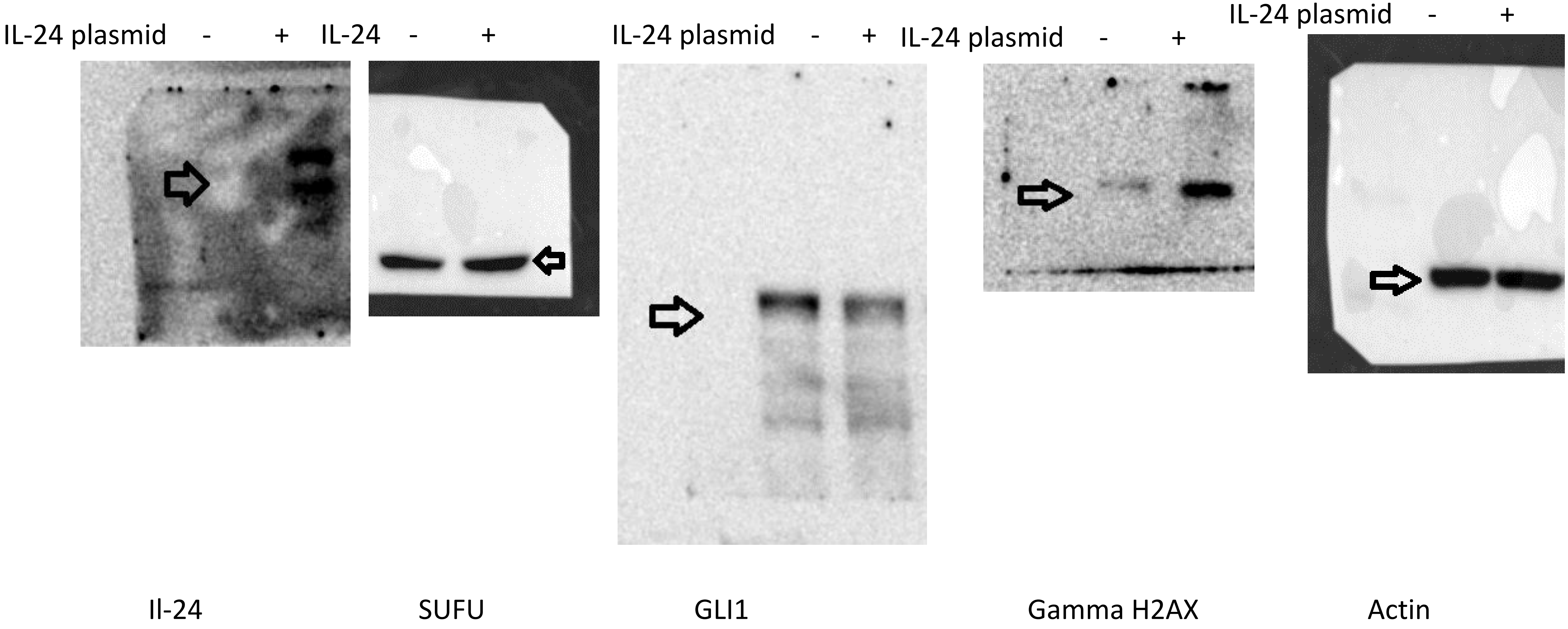

Figure S3 (A549)

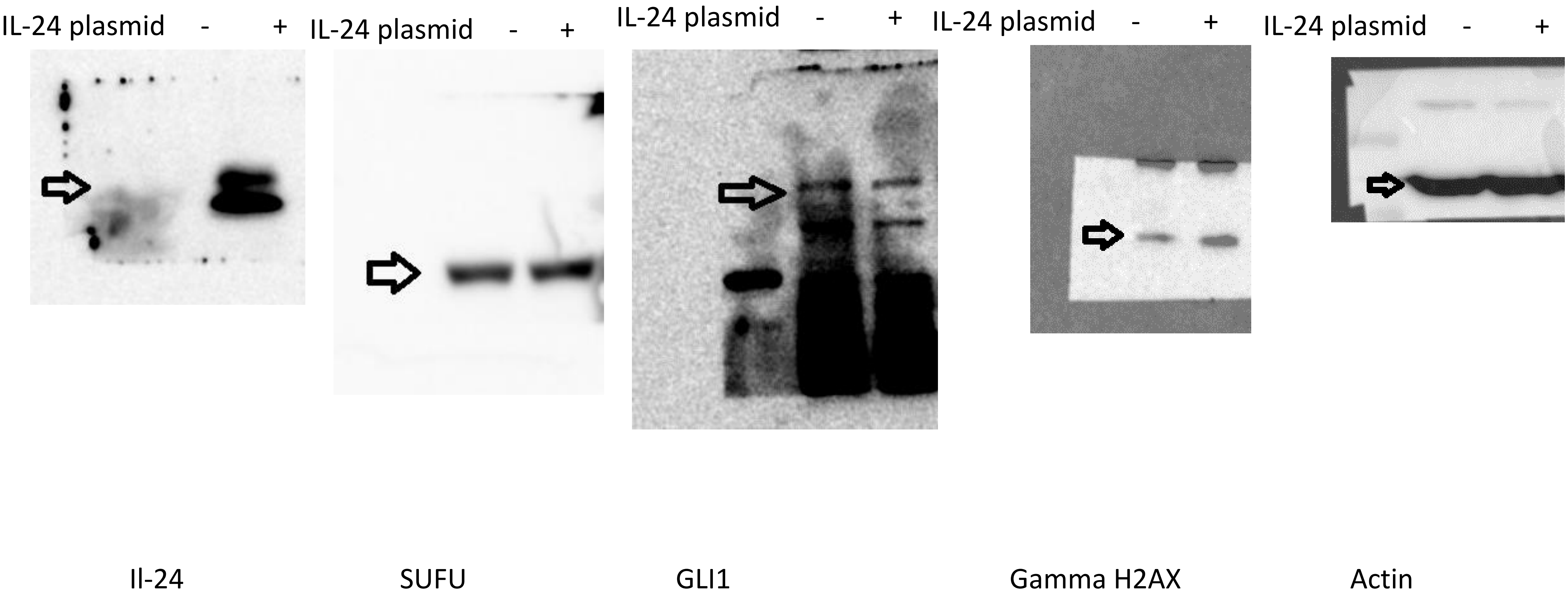

Figure S4

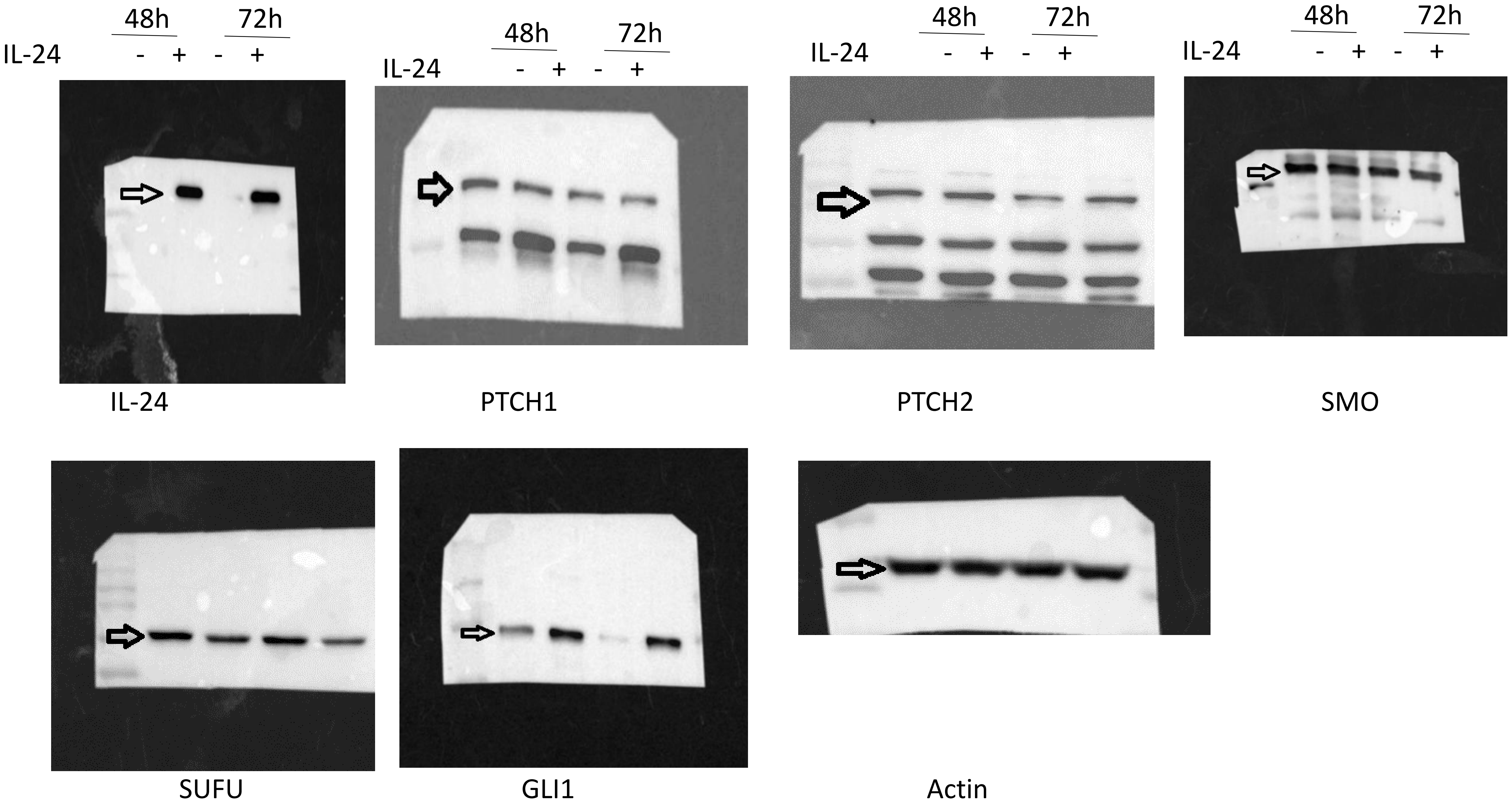

Figure S5

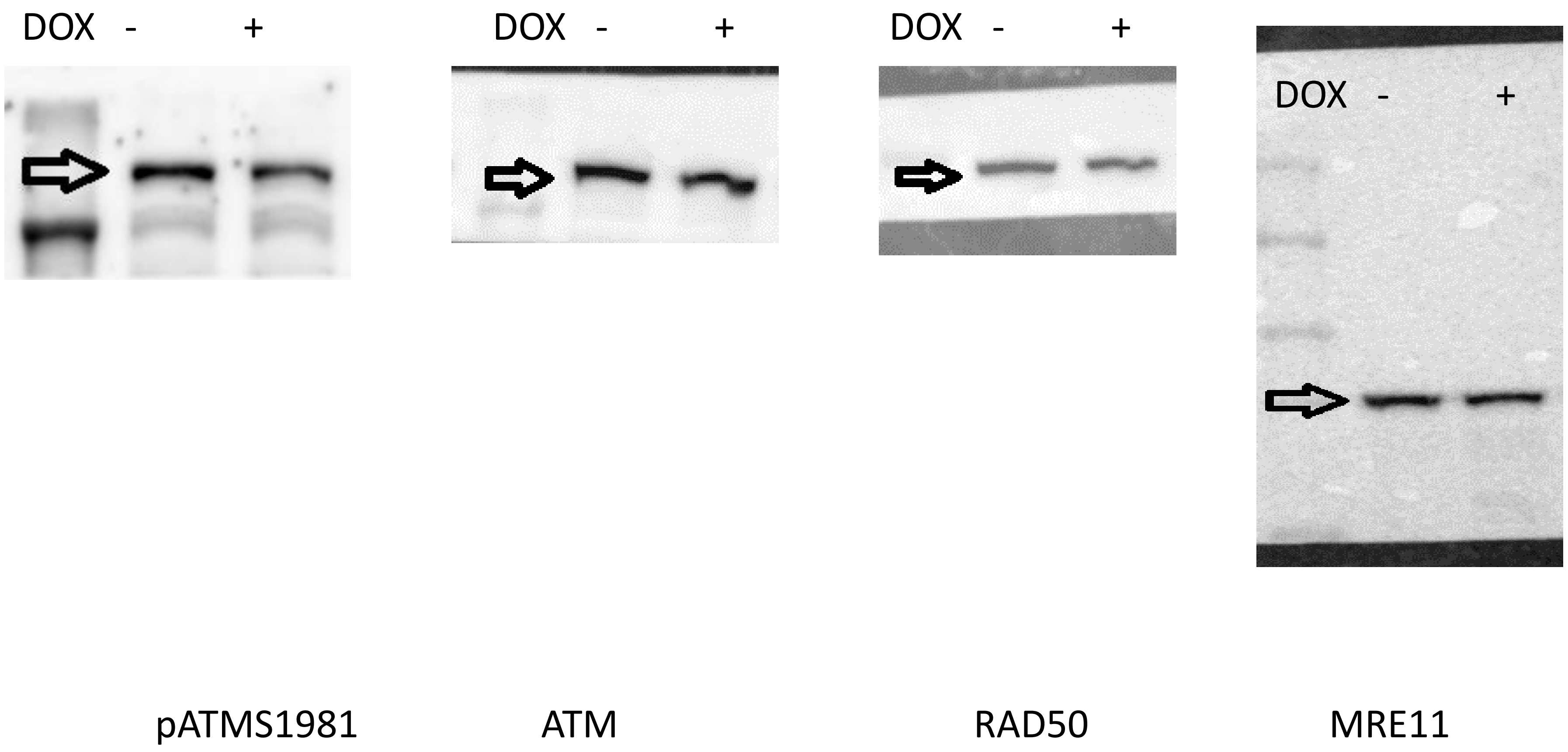

Figure S6

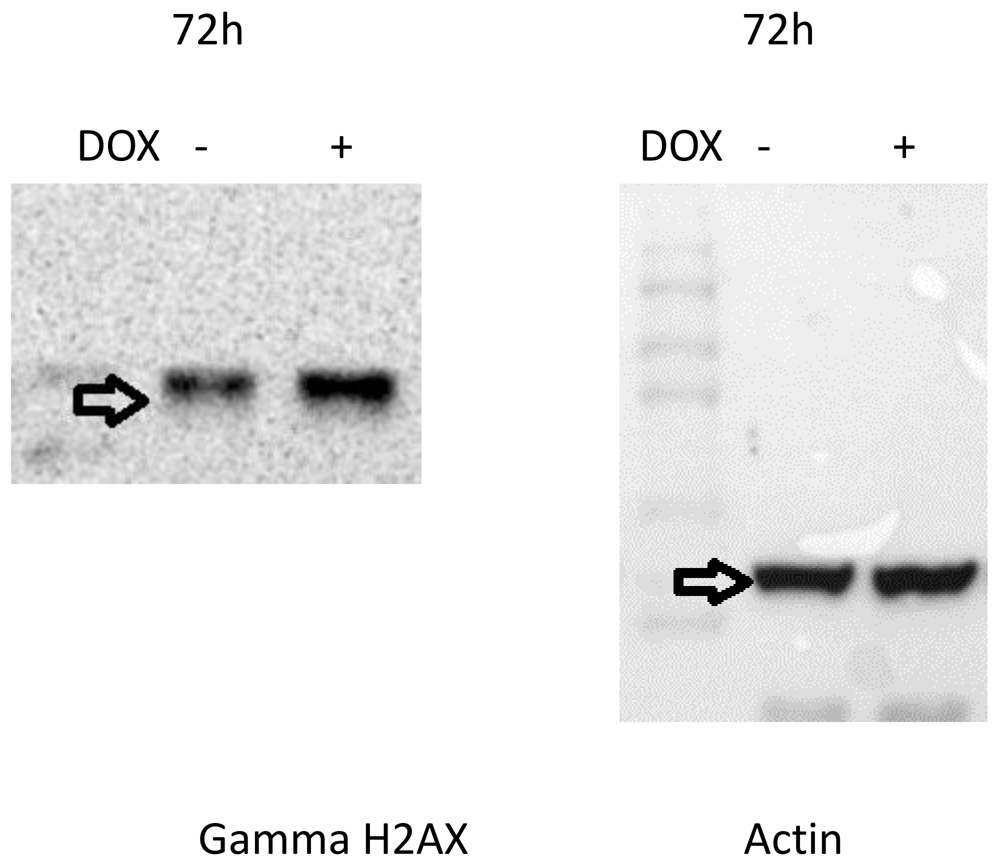

Figure S7

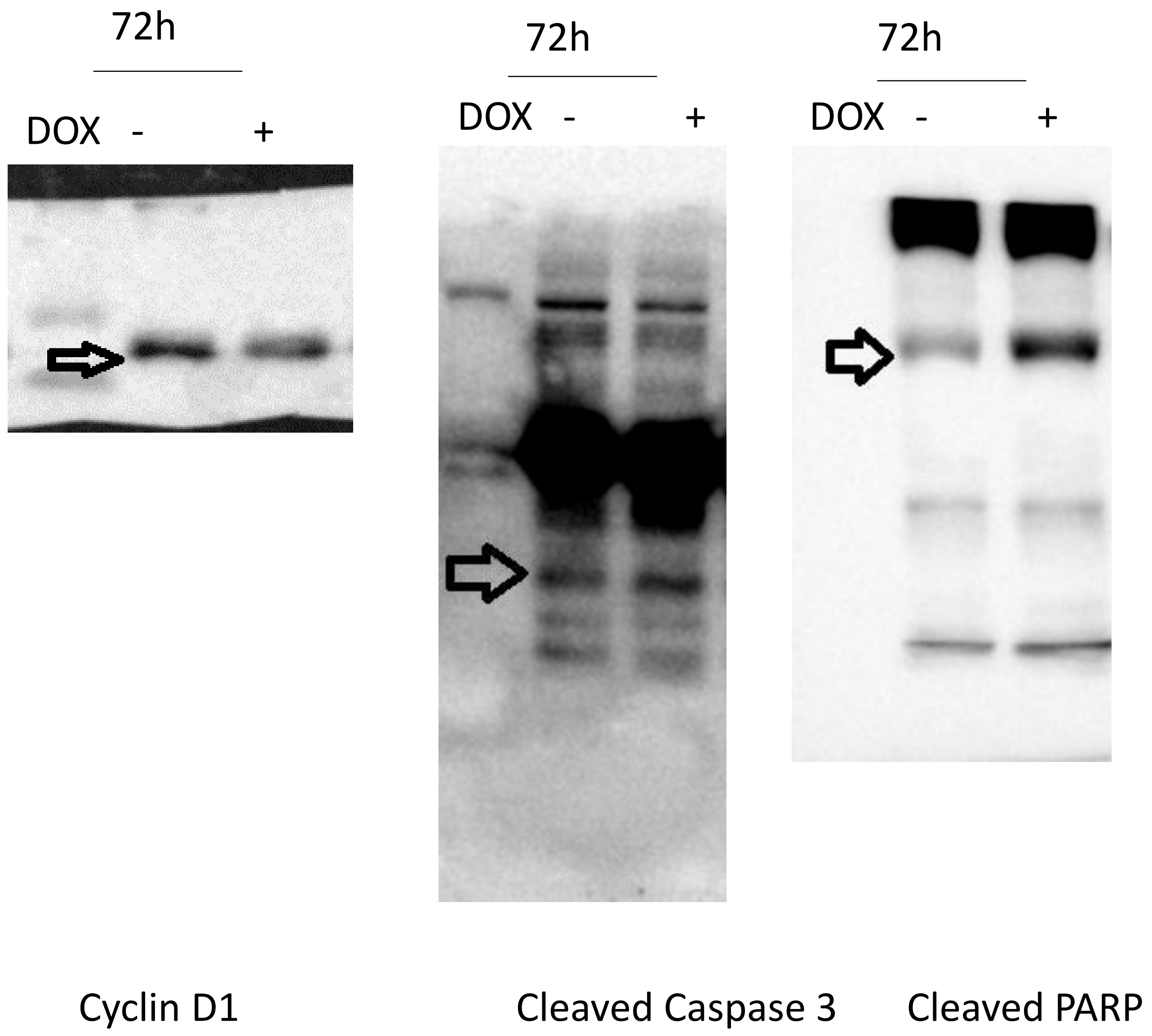
72h
DOX - +
72h
DOX - +
72h
DOX - +
Cyclin D1
Cleaved Caspase 3
Cleaved PARP
